# Chemical Trends in Sample Preparation for Nucleic Acid Amplification Testing (NAAT): A Review

**DOI:** 10.3390/bios13110980

**Published:** 2023-11-10

**Authors:** Soo Min Lee, Hari Kalathil Balakrishnan, Egan H. Doeven, Dan Yuan, Rosanne M. Guijt

**Affiliations:** 1 Centre for Regional and Rural Futures (CeRRF), Deakin University, Locked Bag 20000, Geelong, VIC 3220, Australia; 2 Department of Chemical Engineering, Khalifa University, Abu Dhabi P.O. Box 127788, United Arab Emirates; harikrishnan.balakrishnan@ku.ac.ae; 3 School of Life and Environmental Sciences, Deakin University, Locked Bag 20000, Geelong, VIC 3220, Australia; egan.doeven@deakin.edu.au; 4 School of Mechanical and Mining Engineering, The University of Queensland, Brisbane, QLD 4072, Australia; d.yuan@uq.edu.au

**Keywords:** nucleic acid amplification testing, sample preparation, cell lysis, NA extraction, solid-phase extraction, point-of-need testing

## Abstract

Nucleic acid amplification testing facilitates the detection of disease through specific genomic sequences and is attractive for point-of-need testing (PONT); in particular, the early detection of microorganisms can alert early response systems to protect the public and ecosystems from widespread outbreaks of biological threats, including infectious diseases. Prior to nucleic acid amplification and detection, extensive sample preparation techniques are required to free nucleic acids and extract them from the sample matrix. Sample preparation is critical to maximize the sensitivity and reliability of testing. As the enzymatic amplification reactions can be sensitive to inhibitors from the sample, as well as from chemicals used for lysis and extraction, avoiding inhibition is a significant challenge, particularly when minimising liquid handling steps is also desirable for the translation of the assay to a portable format for PONT. The reagents used in sample preparation for nucleic acid testing, covering lysis and NA extraction (binding, washing, and elution), are reviewed with a focus on their suitability for use in PONT.

## 1. Introduction

Emerging infectious diseases and their potential for worldwide outbreaks have always threatened the global public’s well-being. For example, the recent pandemic was caused by highly contagious pathogens like SARS-CoV-2, which spread exponentially at a growth rate of 0.19–0.29 per day in many countries, burdening healthcare systems and inflicting death and economic damage [1,2]. This outbreak revealed that early and extensive testing for identifying infections enables better and more timely control of the spread of disease. However, conventional detection strategies require a cold chain for sample and reagent preservation and need to be performed in centralized laboratories due to their reliance on advanced instrumentation and skilled personnel. Moreover, the turnaround time of testing is typically several days, and the long interlude costs the time of patients and increases the risk of disease spreading unless appropriately quarantined. For efficient disease surveillance/management, demand for point-of-need tests (PONTs) has increased for wide-ranging applications, including human, animal, and plant health [3,4,5,6].

PONTs are designed to be performed on-site by any user, providing an accurate and rapid (minutes) screening [7]. Microfluidic technologies embedded in lab-on-a-chip devices are typically employed to automate liquid handling of samples and reagents at minute volumes and enable faster and more efficient processing than the macroscale protocols traditionally used in a laboratory setting [8]. For clinical testing of human samples, the World Health Organization called for PONT devices to meet ASSURED criteria: Affordable, Sensitive, Specific, User-friendly, Rapid and Robust, Equipment-free, and Deliverable to end-users, later expanded to REASSURED including Real-time connectivity and Environmentally friendly and Ease of collection [9]. In assessment of the Environmental aspects of an approach, manufacture and disposal of the device and reagents should be considered, including minimisation of the generation of toxic waste at the point of need setting [9].

To diagnose infection with a specific target (e.g., bacteria, viruses), PONT assays and devices employ specific and sensitive molecular techniques, such as immunoassays (IAs) and nucleic acid amplification tests (NAATs) [3,4,6]. While immunoassays have proven effective, NAATs can provide enhanced sensitivity and selectivity as a unique genomic signature is targeted, amplified, and detected. This review focuses on NA tests developed for a PONT setting, abbreviated as NA-PONT. 

Advances in isothermal amplification techniques include loop-mediated isothermal amplification (LAMP) and recombinase polymerase amplification (RPA). These innovations have successfully alleviated the engineering challenges traditionally associated with the gold standard thermocycling polymerase chain reaction (PCR). PCR relies on thermocycling between temperatures of 65 and 95 °C, requiring stringent temperature control. In contrast, isothermal amplification methods operate at a single temperature, typically between 37 and 65 °C. Detailed reviews regarding advanced amplification technologies and associated detection approaches can be found elsewhere [10,11], with the focus of this review on the chemistry of sample preparation. 

Typically, the sample preparation process can be divided into two stages: cell lysis and NA extraction [12]. During cell lysis, membranes of cells and organelles are disrupted to release intracellular components including NAs. Lysis techniques include chemical, mechanical, and thermal lysis, with chemical lysis approaches covering detergents, chaotropic reagents, enzymes, and others. Other reviews have focused on the suitability of different lysis techniques for various sample types [13,14,15,16,17,18,19], and progress towards the integration of lysis techniques in microfluidic devices can be found elsewhere [12,20,21]. While chemical lysis has traditionally been highly effective, carryover of the reagents at 1–10% can cause a complete or significant inhibition of amplification [22,23]. The current review focuses specifically on the reagent composition and trends that may minimize this undesirable inhibition. The section on reagents used for lysis is followed by an overview of the chemical aspects of NA extraction, concentration, and purification. Though liquid–liquid extraction (LLE) has been used, challenges including the need for hazardous solvents (e.g., phenol, chloroform) and time-consuming procedures [24] have driven developments towards the use of solid-phase extraction (SPE)-based approaches. Maintaining the focus on the chemical aspects, this review covers the reagents used for SPE of NAs with anionic and cationic solid phases. Special attention is paid process integration and trends towards rationalising processing steps. The review is concluded by the analysis of a selection of approaches based on the REASSURED criteria, showcasing how the choice of reagents or technology may render an approach more or less suitable for use in low-resource settings. The review is concluded by an analysis of trends that may ultimately lead to faster diagnostics and informed decision making in controlling disease outbreaks. 

## 2. Cell Lysis

Cell lysis is the first step of sample preparation. Its purpose is to release target NAs from biological samples by disrupting the structure of cell membranes, which are also known as phospholipid bilayer membranes or plasmalemma. These membranes are part of the cell’s cytoskeleton and control the transport of materials in and out of the cell, as well as communication with other cells [25]. The upcoming section will analyse various lysis methods that employ detergents, enzymes, alkaline reagents, chaotropic reagents, and other reagents. These methods will be evaluated based on their potential to extract NA for PONT application in chemical perspectives. Additionally, the potential microfluidic platforms for PONT application will also be analysed based on miniaturisation capabilities. A non-comprehensive overview of different chemical lysis approaches reported in the literature is provided in Table 1.

### 2.1. Chemical Cell Lysis

#### 2.1.1. Detergents

Detergents (or surfactants) break down the phospholipid bilayer by virtue of their amphiphilic properties. The membrane solubilisation induced by detergents can be understood in three stages [55,56,57]. Initially, detergent monomers gradually penetrate the outer layer of the membrane, disrupting the orderly arrangement of its molecular architecture. Then, the bilayer becomes saturated with detergent, resulting in phospholipid–detergent mixed micelles. The increasing surfactant content alters permeability and disrupts the osmotic equilibrium of the membrane. This phenomenon forces the detergent-enriched bilayer to fragment and transform into thread-like amphiphilic micelles, leading to complete solubilisation of the bilayer.

Detergents can be ionic and non-ionic, depending on the nature of the polar head. Ionic detergents have charged polar head groups, either positively charged (cationic) or negatively charged (anionic). Cationic detergents often contain ammonium or pyridinium head groups and are used in DNA extraction and cell lysis because the positively charged nature helps disrupt cell membranes and solubilize biomolecules. While anionic detergents, commonly with sulphate or carboxylate ions, are often used in protein electrophoresis, non-ionic detergents have uncharged polar head groups and are suitable for a wide range of applications where ionic interactions should be avoided.

The non-ionic detergent Triton X-100 (2-[4-(2,4,4-trimethylpentan-2-yl) phenoxy] ethanol) was used for cell lysis in a capillary, mixing a 0.1% (*v*/*v*) solution with the sample diffusion owing to the laminar flow regime. Complete lysis of green fluorescence protein (GFP)-expressing cells was achieved within 1 min [58]. In contrast, when *Escherichia coli* (*E. coli*) were incubated in 1% Triton X-100 at room temperature for 5 min, only about 10–15% of viability was observed owing to the stronger bacterial walls and the *E. coli* cell permeability was enhanced to 30% with the aid of additional 1 mg/mL lysozyme [46]. Furthermore, a three-detergent method combining the anionic sodium dodecyl sulphate (SDS), Tween 20, and Triton X-100 (STT) was reported for lysis before RNA extraction from several Gram-negative bacteria, including *Pseudomonas putida*, *Burkholderia cepacia, Agrobacterium tumefaciens*, *E. coli*, *and Edwardsiella tarda*, and Gram-positive *Bacillus subtills* [59]. The quantity of RNA extracted using STT buffer was distinctly greater than single-detergent methods with 2 and 5% SDS, according to the gel electrophoresis analysis. Le et al. investigated a lysis solution containing 0.3% of the non-ionic detergent, IGEPAL CA-630, and 0.1% bovine serum albumin (BSA) to lyse circulating tumour cells (CTCs) [26]. The protocol required a 5 min single step on ice prior to direct reverse transcription (RT)-qPCR to detect RNA from CTCs. The IGEPAL CA-630, octylphenoxypolyethoxyethanol, method outperformed a commercial kit when cell counts were 10 and 100; however, at cell counts around 1000, the higher concentration of RNases degraded target RNA and cell debris inhibited amplification, limiting the effectiveness. The result that detergent-induced lysis can be efficiently performed for low-cell-count samples was also agreed with a buffer containing 0.1% Triton X-100 which was used for 1 min lysis of a single cell [58].

The use of detergents in cell lysis has an impact on different biological samples. Detergents like Triton X-100 are widely used in different concentrations, depending on their specific application. For instance, Triton X-100 concentrations can range from 0.1% for capillary cell lysis to 1% for *E. coli* lysis. In addition, higher concentrations and other surfactants can be used to achieve optimal RNA extraction efficiency in different bacterial species. The concentration of IGEPAL CA-630 varies and has different effects on lysing CTCs based on the cell count. In some cases, a low concentration of 0.1% Triton X-100 is effective for lysing single cells.

**Figure 1:**
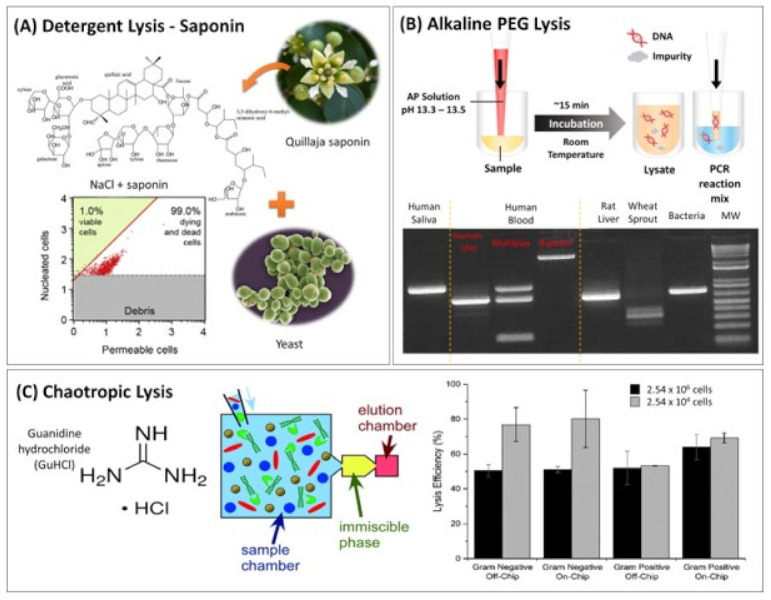
Chemical cell lysis methods. (**A**) A natural detergent extracted from *Q. saponin* for lysing yeast samples. The plot was reprinted from [60] with permission from Elsevier. (**B**) One-step AP lysis method for direct PCR amplification (**top**) and a gel of the PCR product for different sample types (**bottom**). Reproduced from Biotechniques [35] with the permission of Future Science Group. (**C**) Schematic of the 3-chamber DNA purification device (**left**) utilising GuHCl for chaotropic lysis with solid guanidine hydrochloride for lysing bacteria, *H. pylori* (Gram-negative) and *S. aureus* (Gram-positive), from stool samples. On the left, the Immiscible Phase Filtration Assisted by Surface Tension (IFAST) process is shown, used for the extraction of the DNA from the lysate (discussed in Section 3.1.2; the yellow represents the immiscible oil interphase whereas the pink reflects the aqueous elution buffer. The lysis efficiency is compared on and of chip for 2.54 × 10^6^ (black) and 2.54 × 10^4^ cells (grey) (**right**). Reproduced from [41] with permission from the Royal Society of Chemistry.

With increasing interest in developing environmentally friendly PONT assays, using detergents sourced from a natural product in cell lysis was undertaken due to their biocompatibility, environmental sustainability, and adherence to regulatory requirements. For example, saponins from *Quillaja Saponaria*, also known as soap bark tree, were used as a lytic reagent for yeast and combined with NaCl to increase cell membrane permeability by altering the osmotic pressure of the medium to induce plasmolysis (Figure 1A). The viability, expressed as the percentage or fraction of living cells, of *Saccharomyces cerevisiae* significantly decreased from 34.4% in 5% (*w*/*v*) NaCl to 1.0% with the addition of 0.008% *of Q. saponaria* [60]. The lysis effect of saponins on several strains of *E. coli* was visualized using SEM after incubation at 37 °C for a minimum of 1 h [27]. The lytic effect of the natural detergent was not limited to *E. coli*, which is relatively easy to lyse, and was extended to hard-to-lyse yeast. This indicates its potential for versatility in lysis. Therefore, further studies of its compatibility with amplification reactions and various sample types are warranted, with the additional aim of shortening the incubation time, which is ideal for PONT assays.

Although detergent-based lysis is inexpensive and effective for cell lysis, assays using detergents as sole lysis reagents are barely found in NA-PONT applications as the detergent lysis is often slow. The operational time with detergent lysis is usually longer than 1 h under thermal conditions (45–65 °C). Due to its gentle nature, surfactants are often combined with other lysis approaches such as lytic enzymes and/or thermal lysis. Moreover, its lysis efficiency can vary depending on the sample types and the concentration of the amphipaths. Lysis occurs when the concentration of surfactants is close to their critical micellar concentration (CMC) [12,61], and the concentration of the surfactant can be increased if rapid lysis is desired. Excessive surfactant use, however, may lead to bubble nucleation, which may cause practical challenges, including decreased solvent concentration, interrupted electrical and fluidic conductivity, and changes in hydraulic resistance [62,63]. Surfactants can also inhibit the amplification reaction by damaging amplification enzymes due to their denaturing properties effect [46].

#### 2.1.2. Enzymatic Lysis

In enzymatic lysis, a biocatalyst is used to cleave and digest chemical bonds in the membranes. Enzymatic lysis is often combined with detergent for hard-to-lyse samples or samples in a complex matrix to improve the lysis efficiency as mentioned above [28,29,46]. During cell lysis, proteinase K promotes proteolysis to digest proteins and protects the NAs from DNase or RNase, but it requires thermal activation at 50–65 °C to optimize its activity [29,64,65,66]. The HIV virus in human serum was lysed using 1 mg/mL proteinase K and 10 mM dithiothereitol (DTT) mixed with 0.5% SDS and used in conjunction with a paper-based isotachophoresis (ITP) device and RT-RPA. The method allowed for the detection to be as low as 500 copies of viral RNA from 1 mL of spiked serum samples [30]. A similar lysis buffer containing 1% SDS, 10% Triton X-100, and proteinase K (concentration not reported) was used to lyse human adenovirus (HAdV). The recovery rate of the viral DNA was 95% with a limit of detection (LOD) of 10 copies of HAdV in the nasopharyngeal samples collected from infected patients [31].

Lysozymes are routinely utilized in NA extraction kits; however, some pathogens (incl. *S. aureus*) are resistant to lysozyme [67]. Achromopeptidase (ACP), a cocktail of proteases and peptidoglycan-specific hydrolases [68,69], provides an alternative and has been used to lyse Gram-negative bacteria *Bordetella pertussis*, Gram-positive bacteria *Mycobacterium marinum*, and *S. aureus* extensively. As a factor important for PONT, it was also compatible with lyophilisation facilitating storage as a dry reagent. A single, USB-powered platform for bacterial lysis and NA amplification was recently presented using small and large area heaters to deactivate ACP before amplification of DNA specific to methicillin-resistant *S. aureus* (MRSA), respectively [70]. While ACP required thermal deactivation at 90–98 °C prior to amplification due to its inhibitory effect on polymerases [71] like other lytic enzymes including proteinase K, the thermal degradation step was no longer required owing to the immobilisation of ACP on nitrocellulose paper before enzymatic amplification, simplifying the overall workflow [72]. The lysis efficiency on paper was equivalent to that obtained in test tubes. Although ACPs are reported as the broadly applicable enzymes, the direct comparison with proteinase K and/or lysozymes has not yet been found. 

Enzymatic lysis provides effective lysis for hard-to-lyse biological samples and has compatibility with various detergents. Although heat inactivation of lytic enzymes is inevitable for proteinase K before amplification to avoid denaturation of polymerases during the PCR reaction, the enzyme immobilisation technique with ACP made the enzymatic lysis attractive for NA-PONT, with an advantage of enzymatic lysis being that thermal deactivation is no longer required, resulting in a smaller number of sample handling steps. 

#### 2.1.3. Alkaline Lysis

Alkaline lysis (AL) involves the use of high pH to break the fatty acid–glycerol ester bonds in the cell membrane and is often used in combination with a surfactant to aid in the solubilization of the membrane. The first AL protocol was reported in 1979 using a combination of three buffers: Solution I (50 mM glucose, 25 mM Tris-Cl, 10 mM EDTA, pH 8.0), Solution II (0.2 N NaOH, 1% (*w*/*v*) SDS, pH > 13), and Solution III (5 M potassium acetate, glacial acetic acid, pH 4.8) [73,74,75]. The alkaline conditions as a result of the high concentration of NaOH hydrolyse in the phospholipid membranes and subsequent leakage, fusion, and transformation of the lipid bilayer make the membrane permeable [76]. Following neutralisation with potassium acetate, an ethanol-based precipitation of the DNA allows for its isolation. Though the conventional alkaline lysis method can be time-consuming and pH neutralisation is required before amplification [74], AL has been successfully adapted for PONT applications owing to its effective lysis ability for various sample types.

An automated paper-based microfluidic device utilized AL to facilitate on-chip lysis and DNA extraction from small-quantity (1–2 µL) human blood samples. The blood sample pre-washed with 200 µL of DI water was mixed with 10 mM NaOH (no SDS), and after 5 min incubation, 1 mM HCl was used to neutralize the solution, followed by a washing step of the paper with DI water [32]. The automated protocol yielded about an additional 20–40% of DNA compared with a commercial DNA extraction kit, and it was used for DNA extraction directly from various raw samples, including whole blood, buccal swabs, saliva, and cigarette butts, in a process taking less than 8 min. In addition, the extracted DNA had an adequately high quality for downstream analysis with successful demonstration of STR analysis and DNA sequencing. A rapid pork identification method utilized AL of meat products using 0.2 M of NaOH solution. The meat samples (500 mg) were ground up with 4 mL of the NaOH solution and 5 µL of the resultant extract was mixed with 40 µL of the NaOH solution before thermal incubation at 75 °C for 20 min. The lysate was then neutralized using 360 µL of 40 mM of Tris-HCl (pH 7) and 5 µL of the final resultant solution was used for LAMP amplification. This assay allowed for the detection of 0.5 ng/µL of pork DNA and the 0.1% adulteration of pork in beef mixture [77]. The same AL method was compared with the surfactant cetyltrimethylammonium bromide (CTAB) method, which is a common method for DNA extraction from plant samples, and the result of the RPA–Clustered Regularly Interspaced Short Palindromic (CRISPR)/Cas12a assay showed that the lysis effect of the AL with the aid of a 30 min boiling treatment was comparable with the CTAB method, detecting 0.01% (*w*/*w*) pork adulteration [78]. NaOH was used for AL in an assay aiming for the detection of MON863 maize and combined with direct amplification, omitting the extraction and amplification steps. Using a simple 10-fold dilution of the crude cell lysate, MON863 maize was detected after about 8 min of RT-RPA, while the undiluted lysate and its 50-fold dilution attenuated the detection time by 2 min due to inhibition and dilution, respectively [22,33].

AL is faster than lysis using detergent or enzymes. Using AL with 400 mM KOH, 100 mM DTT, and 10 mM ETDA, 80% of *E. coli* cells were lysed after a 5 min incubation at room temperature, while 1% Triton X-100, 1 mg/mL lysozyme, and their mixture led to only ~30% lysis under same incubation conditions [46]. As speed is important for PONT, this makes AL an attractive option; however, the requirement for neutralisation before amplification may form an operational bottleneck in the development of ideal PONT devices. In the traditional AL method, alcohol precipitation can be considered as another bottleneck due to its process length. In addition, the precipitation is routinely performed with high-speed centrifugation at 4 °C [74], which are unfavourable features for PONT devices, leading to the collaboration of the AL method with SPE approaches.

Chomczynski and Rymaszewski alleviated this neutralisation issue introducing an alkaline polyethylene glycol (PEG)-based (AP) lysis method involving a single step for lysing bacteria, eukaryotic tissue samples, and whole blood, using a single reagent consisting of 60% (*w*/*v*) PEG 200 and 20 mM NaOH or KOH (pH 13.3–13.5) [35]. Samples were mixed with 10 times the sample volume of the AP reagent followed by up to 15 min incubation at room temperature. The alkalinity effect of PEG 200 in the presence of a low concentration of KOH rapidly decreased the pH upon dilution with the PCR reaction mix. The AP cell lysate can be subjected to PCR amplification using only a ten-fold dilution in the PCR reagent. The simple workflow of the AP method and its versatile sample range are schematically described in Figure 1B. The AP reagent was modified to 5% (*v*/*v*) NaOH, 1.25% PEG 200, and 10% PEG 8000 to detect dengue virus present in whole blood [36]. By using 0.8 g of 50 µm glass beads with rotation for 90 s at 1500 rpm, the lysis efficiency was estimated close to 100% with a LOD of 10^2^ PFU/mL using LAMP. Lu et al. demonstrated the RPA–lateral flow strip assay to detect *Phytophthora cactorum* in strawberry and *P. infestans* in potato leaf using a modified AP reagent containing 6% PEG 200 and 0.08% NaOH, and this assay—using a 3 min incubation at room temperature for lysis—was capable of detecting as low as 100 fg and 500 fg of pathogenic DNA, respectively [37,38]. In later work, PEG 200 was replaced with PEG 400 to investigate the alkalinity effect of PEG 400, and optimal lysis was observed when twice the AP volume comprising 60% PEG 400 and 100 mM KOH was mixed with whole blood [39]. Application of the AP method to plant samples was demonstrated using a modified AP buffer containing 50% (*w*/*v*) PEG 4600, 20 mM KOH (pH 13.5), and a 10 mm stainless steel bead to improve disruption of the thick cell walls/membrane of the fungus, such as the invasive forest pathogen *Heterobasidion irregulare*, with the minimum LOD of 19.9 pg/μL by qPCR [40].

AP lysis has streamlined sample preparation for diverse applications, including pathogen detection in plant samples and whole blood. It offers high lysis efficiency and compatibility with various samples and amplification reactions in the absence of neutralisation where pH adjustment can be achieved through dilution with the PCR reaction mix. However, this dilution effect may lead to compromising detection sensitivity. Also, it is essential to note that the alkaline conditions in this method can potentially degrade genomic and plasmid DNA, making careful optimisation of the incubation time necessary [79]. Despite these considerations, the AP method remains a valuable tool for simplifying and expediting sample processing in molecular biology and diagnostic applications.

#### 2.1.4. Chaotropic Lysis

Chaotropic lysis is based on the disruption of hydrogen bonding, impacting the protein structure, and compromising hydrophobic interactions within the cell membrane [80,81]. Chaotropic agents also denature the NA-degrading nucleases [82], protecting the NAs. Chaotropic reagents yield high efficiency in lysis and NA isolation. The most commonly used chaotropic reagents for cell lysis are guanidium hydrochloride (GuHCl) [43] and guanidinium thiocyanate (GuSCN) [47] in combination with ethanol and they can be readily found in the commercially available NA extraction kits. 

**Figure 2:**
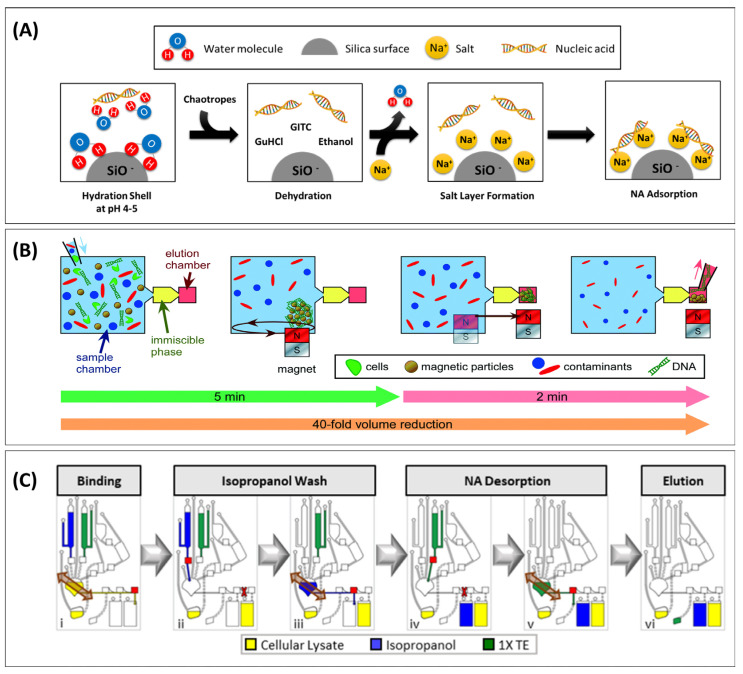
Nucleic acid extraction under chaotropic conditions. (**A**) Schematic overview of nucleic acid binding to an anionic stationary phase in the presence of guanidinium thiocyanate (GTC), a chaotropic agent, showing how Na^+^ ions bridge the anionic charges of the stationary phase and nucleic acid. (**B**) Removal of the chaotropic reagent using immiscible phase filtration, moving magnetic beads (MBs) through immiscible solvent barriers. Reproduced from Ref. [41] with permission from the Royal Society of Chemistry. (**C**) Functional element in multiplexed centrifugal device for solid-phase purification of nucleic acids. Following incubation of the lysate with magnetic particles for binding (**i**), the supernatant is removed (**ii**) before beads are washed in the extraction chamber containing isopropanol (**iii**). TE buffer flows into the extraction chamber (**iv**) and the beads are magnetically actuated to elute NA from the beads (**v**). The purified NA sample is driven into the elution chamber (**vi**). The red squares are laser-activated valves. Reprinted with permission from [45]. Copyright 2021. American Chemical Society.

As shown in Figure 1C, solid GuHCl was used as a sole reagent to lyse liquid stool samples following its dissolution to 5 M in an assay aiming for the detection of *E. coli* and *Helicobacter pylori* using magnetic bead (MB)-based SPE on a microfluidic device (Figure 2A) [41]. Following 5 min incubation at room temperature, the lysis efficiency reached up to 50% for *E. coli* and 60% for *H. pylori.* On average 59.3 and 109.5 ng/µL of DNA was obtained from clinical stool samples using devices with three chambers and five chambers, respectively. In another report, 4 M of GuSCN was added in a lysis buffer containing 20 mM Tris-HCl (pH 7.7), 1 mM DTT, and a redox reagent to aid in the degradation of disulphide bonds, to permeate processed meat samples for the identification of adulteration by qPCR. The paper-based test showed a detection sensitivity as low as 0.1% (*w*/*w*) of pork, beef, and chicken in the samples [42].

Advantages of chaotropic lysis include the fact that it can be performed at room temperature using a short incubation time (e.g., 5 min) and that it can aid in binding the NAs to a stationary phase for NA extraction [41]. However, the appeal of guanidinium salts for lysis is limited by the non-sustainable synthesis, the known inhibition of amplification enzymes requiring additional clean-up, and the hazardous nature that complicates the disposal of PONT devices employing the guanidinium salts, as discussed in more detail below. 

Chaotropic agents are also compatible with enzymatic reagents and/or surfactants to enhance the lysis efficiency. For instance, human saliva was incubated at 56 °C for 30 min in a buffer consisting of GuHCl and proteinase K (concentration not reported) followed by an RNase treatment to quantity bacteria, yielding 157.2–165 ng/µL, or a total DNA recovery of 7.86–8.20 µg [43]. A cell lysis buffer containing GuSCN, proteinase K, and ethanol was also reported, employing a 10 min incubation at 56 °C to detect Gram-negative bacteria by qPCR. Using *Salmonella enterica serovar Typhimurium*, from human urine and fresh milk samples, comparable outcomes to a commercial kit were obtained, reporting a LOD of 5 CFU/10 mL from both sample matrices [44].

The surfactant Triton X-100 is also compatible with chaotropic agent salts for lysis. For instance, 22.92 g GuHCl (equivalent to 6 M), 2% Triton X-100, 0.15 g EDTA, and 0.025 g NaCl dissolved in water giving the final volume of 40 mL facilitated the lysis of Hepatitis B virus (HBV)/Hepatitis C virus (HCV) spiked in human serum samples and yielded 1.3–2.0 µg viral RNA from 100 µL serum spiked with 1000 IU of HBV or HCV via an automated integrated instrument for MB-SPE [2]. The same lysis buffer was adapted into a sample-in-digital-answer-out system to quantitatively detect the pathogenic *Mycobacterium tuberculosis* (MTB)*,* from human serum and saliva samples. This automated system recovered 89.4% and 79.6% DNA from spiked saliva and serum, respectively, and the assay detection limit was 15 to 35 ng/µL MTB genomic DNA (gDNA) depending on mixing [48]. Tween-20 was combined with 1.5 M GuHCl, 50 mM Tris (pH 8), 100 mM NaCl, and 5 mM EDTA, achieving cell lysis in 10 s at room temperature to detect the targeted viral gene in fish blood. In combination with PCR, a LOD of up to 10^4^ cells was presented, comparable with the performance of a commercial kit [49]. The lysis effect of buffers containing GuHCl and 50 mM Tris (pH 8.0), 0.5% (*v*/*v*) Triton X-100, and 1% (*v*/*v*) Tween 20 was tested for cucumber mosaic virus (CMV) [83], demonstrating an increase in recovered viral RNA from 10^5^ to 10^7^ RNA copies with increasing GuHCl concentration from 400 mM to 2 M, but the recovery dropped back to 10^5^ when the concentration of GuHCl was increased further to 4 M, suggesting GuHCl-driven inhibition. Interestingly, a recent study reported that a small amount (40 mM) of GuHCl can significantly improve the turnaround time (10 min faster) of colorimetric LAMP for the detection of SARS-CoV-2 [84]. However, the increased amplification time using 80 mM suggests that GuHCl can only be used in low amounts without washing.

GuSCN was also used with Triton X-100 for combined lysis and extraction in a solution containing 4.8% GuSCN, 5% Triton X-100 in 50 mM HEPES, 4-(2-hydroxyethyl)-1-piperazineethanesulfonic acid, buffer (pH 6.8), and 2 mg of MBs [50]. Combined with qPCR, the LOD was 5 HBV viral particles in 50 µL whole blood. Using slightly lower concentrations of 1% Triton X-100 and 4 M GUSCN and other reagents including 10 mM 2-ethanesulfonic acid (MES) and 1% ß-mercaptoethanol, the lysis of SARS-CoV-2 virus from clinical nasopharyngeal swabs was realized under vigorous orbital shaking at 900 rpm for 5 min. This protocol allowed for the detection of 10 RNA copies/µL, comparable with a commercial kit [51].

Although the chaotropic agent-based lysis buffers have been broadly applied to lyse samples in complex matrices due to their multiple functions in cell lysis and NA extraction, the high concentration of chaotropic reagents imposes a risk of attenuated amplification, and processing steps are needed to remove reagent residues. Additionally, the environmental aspects in synthesis and disposal decrease the appeal for PONT use. 

#### 2.1.5. New Reagents for Cell Lysis

Antimicrobial peptides (AMPs) are small, cationic, amphiphilic molecules that can permeabilize cell membranes of a broad range of microbes, and hence they are promising lytic agents. A study of the use of AMPs for the lysis of hard-to-lyse bacteria, *S. typhimurium* and *S. aureus,* systematically correlated wall structure and AMP activity [85]. AMPs including melittin, magainin analogues (MSI), bombolitin, and cecropin were utilized for lysing bacteria cells in urine samples prior to LAMP [46]. As is shown in Figure 1 of ref. [46], the viability of *E. coli* reached 0% after the addition of 50 µM of different AMPs (cecopin P1, SB-37, MSI-78, and MSI-594) and 5 min of incubation at room temperature, while no lysis of *E. coli* was found with melittin or bombolitin III under this condition. However, most AMPs tested severely inhibited amplification by LAMP, except for the cecropins (P1 and SB-37). When performing LAMP directly from crude bacteria lysate with cecropin P1 treatment, the time to positive improved six times compared to untreated or heat-treated samples.

Ionic liquids (ILs) have unique solvating properties and have also been used for lysis of white blood cells and used for the extraction of NAs without significant interference with amplification. ILs are salts with a melting point < 100 °C and hence are liquid at room temperature. Magnetic ILs (MILs) are a subclass of ILs that include a paramagnetic ion [86]. The hydrophobic MIL trihexyl(tetradecyl)phosphonium tris(hexafluoroacetylaceto)nickelate (II) ([P_6,6,6,14_^+^] [Ni(hfacac)_3_^−^]) and IL ([P_6,6,6,14_^+^] [NTf_2−_]) were used to lyse different plant species (e.g., *Arabidopsis thaliana* and *Nicotiana benthaminana*) within 30–60 s without an additional lysing reagent or heating. Owing to their solvating properties, the NAs were extracted into the MIL with the loaded MIL retained with the help of a magnet allowing for removal of the sample matrix and introduction of the amplification reagents. The MIL facilitated the extraction of 0.5–4 µg DNA from 0.5 mg plant tissue, more than the maximum of 0.6 µg when using [P_6,6,6,14_^+^] [NTf_2−_] [54]. The MIL was compatible with the amplification, attenuating amplification by only 7.9%.

Emerging lysis methods using AMPs and ILs have offered remarkably rapid lysis processes (30 s–5 min), making them attractive for PONT. However, AMP methods have only been used with bacteria (*E. coli*) which are typically fast to lyse; hence, further testing on more sample types is desirable. ILs also allow for fast lysis and provide a greener alternative to many organic solvents, but the high viscosity [87] and cost of ILs [88] mean that further research is required to enhance their appeal for PONT.

### 2.2. Other Cell Lysis Methods

In the early development of microfluidics NA-PONT systems, the focus was to demonstrate amplification and detection, with sample preparation mostly conducted off-chip using commercial kits or instruments. With time, sample preparation protocols have been purpose-developed for PONT use and combined and integrated with NA-PONT systems. Because most of the chemical lysis methods discussed above come with the risk that carryover reagents attenuate amplification, reagent-free approaches including mechanical and thermal lysis methods provide an attractive alternative. A brief overview these chemical-free lysis approaches applied for NA-PONT is provided below. 

For example, a stand-alone miniature and battery-operated bead beater, the Omnilyse, was demonstrated to provide similar performance lysing bacteria cells to the benchtop benchmark Biospec Mini Beadbeater [89]. The instrument remains commercially available more than a decade after its introduction, and it has been used in conjunction with PONT testing, including for the lysis of *Mycobacterium tuberculosis* in sputum [90] and *Chlamydia trachomatis* in vaginal swabs [91]. An overview of reports on acoustic, piezoelectric, thermal, and electrical lysis relevant to NA-PONT is provided below, with more detailed reviews on mechanical cell lysis methods published elsewhere [92,93].

Acoustic forces can be used for lysis, as the interaction of the sound waves with a liquid medium induces rapid streaming flows that can impart shear stresses on the suspended particulate matter including cells, to the point at which the cell membrane is disrupted. Acoustic lysis is effective for mammalian cells but can be more challenging for bacteria, despite an early report in 2005 demonstrating lysis of *B. subtilis* spores with 50% efficiency following 30 s of sonication with a 2.5 μL volume [94]. Its potential for PONT was demonstrated by a comparison between a sonication probe in a cup and channel with the Bulk Acoustic Wave (BAW)-based lysis of *E. coli* demonstrating 50% lysis in 20 s, using 365 times less energy for the channel than for the cup-based approach [95]. Similarly, the use of a traveling Surface Acoustic Wave (SAW) only resulted in an *E. coli* lysis efficiency of 20% of that of surfactant-based lysis [96]. Cavitation microstreaming employs an acoustic field to vibrate an air bubble trapped in a liquid medium, creating frictional forces at the air–liquid interface that generate a circulatory bulk flow that is experimentally relatively simple to apply. Kaba et al. used cavitation microstreaming for lysis of mammalian cell lines in a purpose-designed microchamber with cavities by attaching a piezoelectric transducer to the microfluidic device, using MBs to bind the freed NAs [97]. Under un-optimized conditions, the performance of the device was just under that of commercially sourced kits; however, this was conducted in half the time with less handling and a dynamic range covering five orders of magnitude. Based on theoretical considerations and simulations [98], Zupanc et al. demonstrated hydrodynamic cavitation on the inactivation of bacteriophage phi6 using cavitation [99] with good integrity of the viral RNA. While showing some potential, these results were obtained at the mm scale with high flow rates aiming for disinfection rather than PONT.

The piezoelectric actuation of micropatterned silicon impactor chips in PDMS devices was used to perform cell lysis by physically breaking microbial cell walls via micromechanical impaction. Despite demonstrated efficacy for mammalian cells, more robust and smaller pathogens typically targeted in PONT are more challenging to lyse using this approach. Different silicon microarray geometries and fabrication technique approaches were compared for the efficacy of lysing two yeast species (*S. cerevisiae* and *C. albicans*) to evaluate their efficacy [100]. Despite the effective crushing of beads, the lysis efficiency was estimated < 10% for both species, with future work planned for the optimisation of flow and actuation rates.

As heating is typically required for most NA amplification approaches, thermal lysis appears as an attractive approach. Indeed, a PDMS device was integrated with a carbon paste pad for resistive heating and used for the lysis of Gram-negative *Pseudomonas aeruginosa* and Gram-positive *B. megaterium*; however, the lysis efficiency was not quantified [101]. An attractive tube-based method was reported, showcasing the lysis of *M*. *tuberculosis* and amplification by helicase-dependent amplification in a single heat incubation step at 65 °C for 60 min; the lysis efficiency was similar to chemical lysis, as quantified through culturing plates [102].

During electrical lysis, the cell membrane is opened by exposing it to a high electric field, leading to the release of the intracellular components. Like other physical lysis methods, the appeal of electric lysis for NA-PONT includes the simple operational setting and no need for reagents. However, for small cells such as bacteria (approximately 1 μm long and 0.5 μm thick), the required electric field to achieve the necessary transmembrane potential for lysis (∼1.5 V) is extremely high (>15 kV/cm), requiring, for example, pulsing regimes to allow for heat dissipation [103]. Electroporation of bacterial cell walls was achieved by applying a low-frequency alternating current (AC) field across interdigitated electrodes, demonstrating highly effective bacterial lysis at 0.5 μL/min, with the efficiency dropping at higher flow rates [104]. *Mycobacterium smegmatis* was captured onto a packed bed of microscale silica beads and lysed under an ultrahigh intensity (up to 8000 V/cm) [105]. Using electric pulses, lysis was quantitatively assessed using the mRNA copy number per cell for four representative mRNAs in the cell lysate, with the optimum obtained for 30 pulses in 3 min. Overall, electrolysis provided a significantly more complete release of intracellular mRNAs than bead beating, releasing up to 18 times more RNA molecules. Based on the yield dropping off for higher voltages, the authors concluded that the lysis was near quantitative, but the efficiency was not calculated.

Electrical lysis was combined with electrophoretic concentration of bacteria on a nanoporous membrane, using the high potential drop across the membrane for lysis of the concentrated bacteria [106]. The efficiency of the device was determined through bacterial culture of the lysate and was found to be 90% when a potential of 300 V was applied for 3 min. While qPCR was conducted to confirm the quality of the DNA from the lysed cells, further work preventing loss due to non-specific binding and methods to collect the DNA from the lysate are required for interfacing this approach to NA-PONT.

The high field strength demand for electrical lysis was mitigated by combining electrical lysis with mechanical lysis, using ion concentration polarization (ICP) near ion-selective membranes (ISMs) for the formation of fast electro-convective vortices concentrating agitated bacterial cells toward the high field region near the ISM walls [103]. A low electric field (100–300 V/cm) enabled bacterial lysis even in physiological buffer (e.g., 150 mM). While the high (>88%) protein yield demonstrated efficient lysis, the mRNA recovery was only 5%, but it was still better than that obtained using control experiments using bead beating.

In conclusion, reagent-free fluidic approaches have demonstrated good efficiency for the lysis of mammalian cells; however, bacterial samples have proven more challenging. Building on the progress made, however, we are confident that chemical-free lysis will continue to expand, driven by the desire minimize the reagents used in the NA-PONT workflow.

## 3. Nucleic Acid Extraction

### 3.1. Solid-Phase Extraction (SPE)

SPE is based on the selective binding of a target to a solid support, allowing for the removal of unbound interferences using washing agents that maintain the binding conditions, followed by elution of the target off the solid support under conditions where the target no longer binds the support. During elution, the bound NAs are mobilized [81,107,108], and the collection of the target into a volume smaller than the original sample volume can be used for concentration enhancement. NAs can be considered a polyelectrolyte, with the phosphodiester backbone providing negative charge. Electrostatic interaction is therefore the main mode of binding the DNA to the support. One of the most common stationary phases used for SPE is fused silica, containing weakly acidic silanol groups (-SIO^-^) that are negatively charged when the pH is greater than 4 [81,109]. Chaotropic and non-chaotropic reagents can be involved in the facilitation of the electrostatic binding on anionic supports and are summarized in Table 2 and Table 3, whereas reagents used for SPE on cationic supports are summarized in Table 4. These tables were composed focusing on the chemical aspects, with fluidic handling and processing discussed later. Details of the lysis approach are also included, noting that only chaotropic, AL, and mixed approaches like detergent/enzyme mixtures provide effective lysis in minutes and hence are suitable to be used in combination with the extraction approaches developed for PONT.

Early NA-SPE devices were designed to accommodate methods that directly replicate the protocols from commercially available NA extraction kits, and while the exact composition of the reagents may not be disclosed, binding reagents may include components that can inhibit amplification [22,41,110] and hence need to be washed away before elution. The final step of the SPE is the elution, and re-mobilising the NAs is realized by decreasing affinity to the stationary phase, for example, using an elution buffer with elevated pH (6–8) and low salt for silica-based anionic stationary phases or alkaline buffers for cationic supports [44,49,52].

#### 3.1.1. Reagents

##### Anionic Supports under Chaotropic Conditions

One of the most common stationary phases used for SPE is fused silica, containing weakly acidic silanol groups (-SIO^−^) that are negatively charged when the pH is greater than 4 [81,109]. The phosphodiester backbone means that DNA is an anionic polyelectrolyte. The electrostatic repulsion between negatively charged NA and SIO^−^ can be mitigated by concentrated salts and chaotropic agents (e.g., GuHCl, GUSCN, ethanol) [108], also changing the helical structural configuration of B-DNA to either the A- or C-DNA forms, both less favourable to binding water [81]. The chaotropes also remove surface-bound water from the silica surface, weakening non-covalent interactions (e.g., hydrophobic interactions, van der Waals force, hydrogen bonding) and overall reducing the hydration [81,111]. The chaotropic decrease in length scale for electrostatic interactions decreases the energetic penalty normally involved with electrostatic repulsion, while the lowered free water content energetically favours the non-covalent binding of the NA to the support. The chaotrope-facilitated binding is illustrated in Figure 2A, recognising that the binding can also be facilitated by cationic chaotropes where no additional salt is required. A non-comprehensive overview of PONT testing approaches using extraction under chaotropic conditions is provided below and summarized in Table 2.

**Table 2:** Nucleic acid extraction on anionic stationary phases using a chaotropic agent for binding.

Solid Phase	Surface	Binding Buffer	Washing Buffer	Elution Buffer	Elution Volume	Target	Sample Matrix	Amplification	LOD	Ref.
MB	Silica	Ethanol	Kit	Kit	10 µL	HPV virus	Synthetic DNA	qPCR	-	[19]
MB	Silica	GuHCl	Mineral oil + GuHCl	Water	10 µL	Bacteria	Liquid stool (clinical)	PCR	-	[41]
Glass membrane	Whatman glass pad	20 mM Tris-HCl, 4 M GuSCN, 1 mM DTT, pH 7.7 (lysis buffer)	Isopropanol, 15% *v*/*v*	Water	5 µL	Animal	Mixed meat (minced)	qPCR		[42]
Paper/Disc	Cellulose	1.5 M GuHCl, 50 mM Tris [pH 8], 100 mM NaCl, 5 mM EDTA, 1% Tween-20	10 mM Tris pH 8.0, 0.1% Tween-20	Water	10 µL	Viral gene in fish	Blood (fish)	PCR	10^4^ cells	[49]
MB	Silica	GuHCl, TRIS, EDTA, NaCl in ethanol (50%)	Wash buffer I (GuHCl + 68% *v*/*v* ethanol) Wash buffer II (70% *v*/*v* ethanol)	10 mM Tris, 0.1 mM EDTA pH 8	200 µL	Bacteria	Serum and saliva	RPA	-	[48]
MB	Silica	GuSCN + Triton X-100 (pH 6.8)	Organogel (12-HAS) GuSCN (pH 6.8) Ethanol NaCl2 (pH 7.6)	10 mM Tris, 0.1 mM EDTA pH 8 (TE buffer)	100 µL	Virus	Spiked blood	qPCR	5 particles	[50]
MB	Silica	4 M GuSCN, 10 mM MES (2-ethanesulfonic acid), 1% Triton X-100, with 1% ß-mercaptoethanol	oil immersed, Ethanol, 50% *v*/*v*, then water Water	None (on-bead amplification)		Virus	Nasopharyngeal swab	LAMP	1–10 copies/µL	[51]
MB	Silica	3 M GuHCl, protein kinase K, at elevated T	Isopropanol	10 mM Tris, 0.1 mM EDTA pH 8	8 µL	Virus	Buccal swab	LAMP, qPCR	-	[45]
MB + Steel wool	Silica	GuSCN + EDTA + Tris-HCl + Triton X-100	GTIC Ethanol Tris-HCl + EDTA		30 µL		Synthetic sputum + residual urine sample	qPCR	-	[52]
MB	Silica	Isopropanol	Washing buffer 1 and 2	Elution buffer		Virus	Cervical swab	PCR	10^3^ copies/mL	[112]
MB	Silica	3.5 M GuSCN, isopropanol, 45% *v*/*v*, 2.5% Tween 20, 10 mM Tris pH 8.0, 1 mM EDTA	3 M GuSCN isopropanol 30% *v*/*v*, 5% Tween 20, 40 mM Bis-Tris pH 6.0, 2 mM EDTA then 50 mM Tris pH 8.0 0.5 mM EDTA ethanol 80% *v*/*v* then ethanol, 100% *v*/*v*			human	Plasma	PCR	-	[113]
GB	Glass for DNA, oligo(dT) functionalized for RNA	Ethanol	Fluorinated oil Buffer AW1/2 (DNA) or Tris-HCl (pH 7.5) + LiCl + EDTA (RNA)	DNA: Tris HCl, 10 mM, EDTA, 0.5 mM, pH 9/RNA, Tris-HCl (pH 7.5)	100 µL	mRNA	THP-1 cell	qPCR/RT-qPCR	10 cells	[110]
MB	Selective recognition using NA probe		100 mM phosphate, 150 mM NaCl, pH 6.0	10 mM Tris, 50 mM NaCl, pH 8.0	10 µL	Virus	Lab culture	Amplification-free	0.021 pfu/mL	[114]
Membrane (kit, ground)	Silica	Buffer AW	Buffer AW1/2	Kit		Virus	Lab culture	RT-LAMP	25 copies	[115]
Porous silicon	Silica	6 M GuSCN in 10 mM Tris, 1 mM EDTA (pH 8) with 1% Triton-X 100 (adjusted to pH 6.4)	Ethanol, 70% *v*/*v* in 10 mM NaCl	10 mM Tris, 0.1 mM EDTA pH 8 (TE buffer)	10 µL, 25 µL					[116]
Silica filter	Silica	Binding buffer	GuSCN,3 M 25% *v*/*v*, Ethanol, 75% *v*/*v* then Ethanol 96% *v*/*v*	Water	50 µL	HPV 16	Cervical specimens	NASBA		[117]
Paper	Polyether sulfone (PES)	GuSCN, NaCl, 1-butanol, glycoblue (coprecipitant)	Ethanol 70% *v*/*v*, then 100% *v*/*v*	LAMP mix	12.5 µL	HPV 16	Cervical specimens	LAMP	1.2.10^5^ copies	[118]
Paper	Paper also polymer monolith	2.6 M GuSCN, 300 mM NaCl, 35% *v*/*v* 1-butanol 45 μg glycoblue (co-precipitant)	Ethanol 70% *v*/*v*, then 100% *v*/*v*	Tris EDTA	200 µL	Bacteria	Synthetic urine	Isothermal helicase-dependent amplification (tHDA)		[119]

As mentioned in Section 2.1.4, chaotropic reagents can also play a role in lysis, often making it a dual-purpose reagent [120,121]. The multiplexed centrifugal microfluidic device for NA-SPE utilized different concentrations of GuHCl for lysing viral samples from buccal swabs and binding SARS-CoV-2 RNA (Figure 2B). For the lysis, 6 M GuHCl with 10 µL proteinase K was introduced to a swab cutting. After lysis, a suspension of MP in 3 M GuHCl was employed to capture RNA during 8.5 min incubation [45] (Figure 2C). The previously mentioned microfluidic device for on-chip NAAT of *Helicobacter pylori* from stool samples facilitated simultaneous cell lysis and DNA binding within 5 min by reconstituting solid GuHCl in presence of silica MPs [41]. Lysis/binding buffers that consisted of 4.8% GuSCN, 5% Triton X-100, and 2 mg of magnetic particles (Dynabeads MyOne Silane) were used to extract hepatitis B virus (HBV) DNA from blood [50]. A lysis/binding buffer with similar composition (4 M GUSCN and 0.5% Triton X-100) was used to detect MTB. In combination, 2 mg silica paramagnetic particles (PMPs) and 17 ± 1 mg steel wool as a ferromagnetic matrix enabled the capture of the MPs on the wool during a 3 min incubation at room temperature, recovering 10.2 ± 4.03% and 91.2 ± 7.46% of target DNA from sputum and urine, respectively [52].

Apart from Gu-based chemicals, alcohols, including ethanol, are chaotropic and interfere with hydrogen bonds [122] and as such have been used to promote NA binding/precipitation on the silica or imidazole functionalized carboxyl PMPs [44,48,51]. Isopropyl alcohol (or isopropanol, IPA) was added to enable DNA–silica interaction to detect human papillomavirus (HPV) from cervical swabs using a hand-sized fully integrated microfluidic device [112]. The increased binding efficiency was observed with about a 0.6 cycle improvement in the RT-qPCR test in the presence of 50% IPA and 1.25 M NaCl during the RNA–silica powder (glassmilk) binding process [123]. Binding buffers combining both Gu-based reagents and alcohol are also frequently reported. For example, 500 µL of a binding buffer consisting of 30% (*v*/*v*) IPA, 3 M GuSCN, 5% (*v*/*v*) Tween 20, 8 mg/mL proteinase K, 13 mM Tris (pH 8.0), 4 mM EDTA, and 1% (*v*/*v*) silica-coated superparamagnetic beads was added to immobilize cell-free DNA (cfDNA) from 10 mL plasma through a 5 min incubation, yielding 4.3 ng/mL DNA with an 84% recovery rate [113]. 

Once the NAs are bound to the solid support, unbound compounds can be removed during washing with purpose-designed buffers that keep the NAs electrostatically immobilized. The removal of impurities allows for the elution of the NAs in a clean fraction, ready for amplification. Washing buffers typically resemble the chemical composition of binding buffers and may contain chaotropic salts at reduced concentration to maintain binding, but they may also include alcohols (e.g., ethanol, isopropanol). Washing buffers used in laboratory-based NA extraction kits using silanol surfaces typically contain ethanol (50–100%) [51,85,124] or isopropanol (15–80%) [34,42,45] to remove salts and contaminants while precipitating the NAs [125]. Ethanol and isopropanol have a lower dielectric constant (ε) than water (ε of 24.6 and 18.3, respectively, vs. 78.5 [78]) [126,127], making the nucleic acids less hydrophilic by decreasing ionisation. While the NAs are immobilized at the surface, soluble interferences can be removed with minimal NA loss.

Chaotropic agents are versatile as they can serve both cell lysis and NA binding, but the potential inhibitory effect of the chaotropes during amplification remains a concern. Moreover, the global shortage of GuSCN, which also has storage challenges as a toxic gas, hydrogen cyanide (HCN), can be formed when in contact with acid [128], places a great impetus on the development of alternative binding chemistries without the involvement of these chaotropes [123].

##### Non-Chaotropic Binding

**Figure 3:**
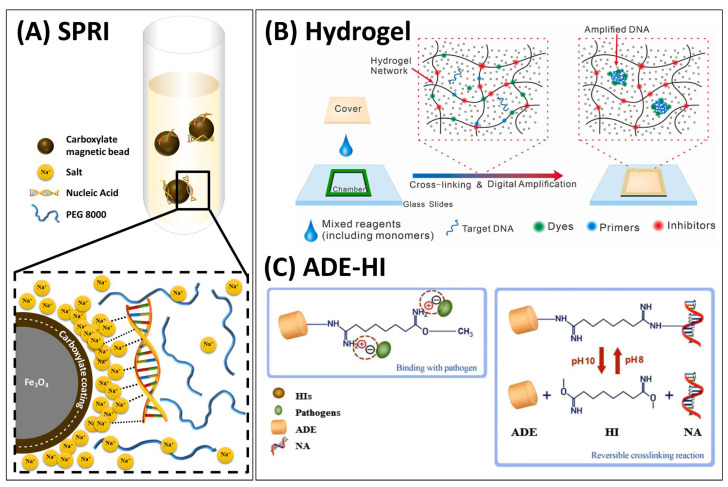
NA isolation under non-chaotropic conditions. (**A**) A schematic illustration of solid-phase reversible immobilisation (SPRI) technique. (**B**) A hydrogel-based DNA isolation for on-gel LAMP detection of bacteria in food samples. Reprinted from [130] with permission from Elsevier. (**C**) A pH-dependent reversible crosslinking reaction in the presence of homobifunctional imidoesters (HIs) during nucleic acid binding on amine-functionalized diatomaceous earth (ADE). Reprinted from [131] with permission from Springer Nature.

The risk of the inhibitory effect of chaotropic reagents has made SPE methods that are not reliant on chaotropes an attractive alternative, and several approaches have been reported to eliminate the probability of chemical contamination with the reagents [31,129]. Non-chaotropic approaches to facilitate DNA isolation have used crowding or crosslinking reagents in the NA-SPE process (Figure 3). A non-comprehensive overview of PONT approaches using extraction under non-chaotropic conditions is provided in Table 3. 

PEG is a macromolecule that induces molecular crowding because solvents, solutes, and polymers are unable to occupy the same space simultaneously. For instance, about 6% (*w*/*v*) of PEG 8000 occupied nearly 40% of the initial volume, while 6% (*w*/*v*) of PEG 4000 and PEG 1000 excluded about 20 and 10% of the volume in a test tube, respectively [132]. The reduced volume enhances physical interaction due to increased free energy of the system by restricting the conformational entropy of molecules [133,134]. The crowding effect of PEG 8000 in SPRI is schematically shown in Figure 3A and a more detailed review of the molecular crowding effect on the structure and stability of biomolecules (e.g., NAs, proteins) can be found elsewhere [134]. Carboxylated paramagnetic beads were used by Hawkins et al. to immobilize DNA samples with different DNA sizes ranging from 7.2 kB to 240 kB, by utilising 10% PEG 8000 and 1.25 M NaCl [135], yielding 80% plasmid DNA. This method was termed as Solid-Phase Reversible Immobilisation (SPRI) and allowed for DNA extraction without Gu-based chemicals, filtration, and centrifugation. Although the SPRI has been predominantly used for clean-up of PCR products [136] or size selection [137] due to its tunability of the affinity based on the size of DNAs by controlling the mix ratio of PEG and salt concentration [138], it can also be used for NA isolation. For example, SPRI-based DNA extraction using 18% (*w*/*v*) PEG/1 M NaCl buffer [136] was demonstrated to process faecal, cloacal, and oral swab samples, yielding much greater (261.12 ± 390.08 ng, 233.52 ± 142.83 ng) total DNA from the cloacal and oral swabs than a commercial MB DNA extraction kit (8.31 ± 11.66 ng vs. 3.38 ± 2.04 ng, respectively) [139]. Instead of a carboxylate coating, DNA immobilisation on non-coated Fe_3_O_4_ PMPs was demonstrated to detect human and bacterial DNA from saliva samples [43]. The binding buffer containing 10% PEG (molecular weight not reported), 2 M NaCl, and 40 µL/mL synthesized magnetic particles was mixed with saliva samples in the preservation buffer (SPB) (0.2 M Tris, 42 mM EDTA, 1 M urea, 1% SDS, and 0.1% azide), recovering 7–16 µg of target DNA from a 500 µL sample through conventional PCR.

For anionic stationary phases under non-chaotropic conditions, washing is typically also conducted with alcohols alone, whereas the elution is conducted in water (Table 3).

**Table 3:** Nucleic acid extraction on anionic stationary phases using non-chaotropic conditions.

Solid Phase	Surface Group	Binding Buffer	Washing Buffer	Washing Steps, Volume	Elution Buffer	Elution Volume	Target	Sample Matrix	Amplification	LOD	Kit vs. Assay	Ref.
**MB**	Fe_3_O_4_ nanoparticles	PEG + NaCl	Ethanol, 75% *v*/*v*		Water	50 µL		Human saliva	PCR	n/a	Assay	[43]
**Glass filter**	Glass	200 mM NaOH with 1% SDS (=lysis buffer)	Isopropanol, 15% *v*/*v*	1, 75 µL	Water	2 µL	Bacteria (aerosol spiked)	Cultured	qPCR	10 CFU	Kit	[34]
**MB**	Silica	Lysis buffer (SDS and Protein Kinase K)	Washing buffer 1 and 2	2	Low ionic strength elution buffer	100 µL	K562, CHO-K1 cells	Culture media	PCR	18 cells	Comparable but faster	[97]
**MB**	Carboxyl (to compare with silanol)	PEG 8000 (18 wt%), NaCl, 1 M	Ethanol, 80% *v*/*v*		Water		Animal	Faecal swab	PCR	35.53 ± 15.03 ng (faecal) 261.12 ± 390.08 ng (cloacal swab) 233.52 ± 142.83 (oral swab) 87.3 ± 7.2% N/A		[139]
**MB**	Silica	5% PEG8000, 0.5 M NaCl, and 3.5 mM KOH	Wash can be eliminated		On-bead amplification	-	Bacteria	Artificial saliva, sweat, urine	qPCR	0.15 CFU/50 μL	Assay	[140]

##### Cationic Supports

Cationic supports have gained increasing popularity, as binding and elution can be realized under milder conditions (no extreme pH or chaotropes) and hence at a decreased risk for attenuating amplification. A non-comprehensive overview of PONT approaches using cationic stationary phases for extraction is provided in Table 4. A cationic poly-L-histidine surface was used for on-chip *Salmonella* DNA purification under continuous flow, showing DNA binding at a pH lower than the pK_a_ of surface amine groups and DNA elution at a pH higher than the pK_a_ value [141]. The recovery efficiency of DNA using an elution buffer at pH 10.5 was >95%, with approximately 87% of the eluted DNA present in the first 70 μL. FeO_4_ nanoparticles coated with the ampholyte polydopamine (PDA) were also used for NA-SPE, showing the best extraction efficiency at 20% PEG, 4 M NaCl, and pH 2 [142]. The PDA-coated nanoparticles allowed for the extraction of DNA with a higher yield and purity than spin column and magnetic bead kits and facilitate the extraction of 117 mg/g human DNA with a 90% yield. The benign reagents allowed for on-bead amplification, eliminating the need for elution. PDA-coated FeO_4_ nanoparticles were also used to immobilize a selective nucleic acid capture probe to quantify DNA using competitive displacement of a fluorescently labelled oligo, without the need for amplification [143]. 

Reversible NA binding to cationic supports can be facilitated by homobifunctional imidoesters, acting as a crosslinking reagent to covalently link the free amino groups of the DNA as well as electrostatically interact with the negatively charged phosphodiester bonds, while also forming covalent amidine bonds with aminated surfaces [144]. As the amidine bonds formed are reversible at high pH (>pH 10), this can be used to control capture and release by changing pH. NA-SPE was reported using dimethyl adipimidate (DMA) on the microchannel walls functionalized with 3-aminopropyltriethoxysilane (APTES) and combined with label-free detection using a silicon micro-ring resonator [145]. The DNA binding efficiency of the micro-chip was improved from 34% to 98% with the aid of DMA. This technique was then integrated into a disposable microfluidic chip for a self-powered switch-controlled NA extraction system (SSNES) and yielded abundant *HRAS* genes from the urine samples. However, the binding efficiency of DMA was uncertain as DNA adsorption occurred in the mixture of the Gu-based commercial lysis buffer. Furthermore, the extraction efficiency of the non-chaotropic agents was tested with a detergent lysis buffer (0.1 M Tris-HCl (pH 8.0), 10 mM EDTA, 1% SDS, and 10% Triton X-100) and the result was improved by replacing DMA with another linking reagent, dimethyl pimelimidate (DMP) [53] and dimethyl suberimidate (DMS), which also captured NAs and formed covalent bonds with the primary amine groups (-NH_2_) [146]. The qPCR analysis showed that the DMP and DMS systems were not only comparable with a commercial extraction kit but also improved the sensitivity of the system by detecting as low as 1 CFU/mL of *Brucella* and 10 viral DNA copies/reaction. A hand-powered NAAT-PONT device was integrated with amine-functionalized diatomaceous earth (ADE) with homobifunctional imidoesters to detect various bacteria from human urine and serum samples (Figure 3C) [131]. When the amine groups of ADE are exposed to HIs (e.g., DMA, DMS, and DMP), reversible links between the amine groups of NAs and ADE are created at pH 8, as illustrated in Figure 3C. Although the NA binding occurs based on the charge interaction between ADE and the negative NA, this binding can be reversed by deprotonation of amine groups with a high-pH elution buffer (pH 10). The DNA capture efficiency was improved by up to 98.3% with a LOD of 1 CFU/mL by up-scaling the sample volume (<50 mL), which was 100-fold greater than that of the commercial extraction kit.

For the cationic stationary phases, washing is typically conducted in PBS, at a pH maintaining the protonated nature of the stationary phase as required for DNA binding (Table 4), whereas the elution of the NAs is realized using a basic eluent, such as a carbonate buffer with pH > 10.

**Table 4:** Nucleic acid extraction on cationic stationary phases.

Solid Phase	Surface	Binding Buffer	Washing Buffer	Elution Buffer	Elution Volume	Target	Sample Matrix	Amplification	LOD	Ref.
Acrylonitrile butadiene styrene (ABS) device	(3-Aminopropyl)triethoxysilane (APTES)	Dimethyl suberimidate in TE-based lysis buffer	PBS	NaHCO_3_ 10 mM, pH > 10		Virus	Clinical	RPA	10 copies	[31]
MB	Imidazole	Tris-HCl (pH3)	PBS	NaHCO_3_ (pH 10.6)		Bacteria	Human urine/milk	qPCR	5 CFU/10 mL	[44]
PMMA	Histidine or polyhistidine	0.5 M KAc, pH 5.0	0.5 M potassium acetate, pH 5.0	NaHCO_3_ (pH 10.6)	10 µL	Bacteria	Culture	PCR	<5000 cells	[141]
Amine-functionalized diatomaceous earth	3-aminopropyl(diethoxy)methyl silane	Dimethyl suberimidate in lysis buffer (Proteinase K, Tris-HCl [pH 8.0], EDTA, SDS, Triton X-100, lysozyme solution RNase-Free Dnase)	PBS	NaHCO_3_ 10 mM, pH > 10	100 µL			PCR qPCR		[131]
Fe_3_O_4_	PDA	PEG, 20% *v*/*v*, 4 M NaCl	Ethanol, 70% *v*/*v*	10 mM Tris-HCl, 1 mM EDTA, pH 8	50 µL			PCR		[142]
Glass slide	APTES	Dimethyl adipimidate (DMA)	PBS	NaHCO3 (pH 10.6)	150 µL	HRAS gene	Urine	PCR	-	[145]
ABS chamber wall	APTES	Dimethyl pimelimidate (DMP) in 100 mM Tris-HCl (pH 8.0), 10 mM EDTA, 1% SDS, and 10% Triton X-100) with either proteinase K (for DNA) or proteinase K and DNase I (for RNA)	PBS	NaHCO3 pH < 10.6	50 µL/min	Viral/bacterial, cancer	Plasma	PCR	1 CFU/mL (10 cells/100 µL for cancerous cells)	[53]
MB	ChargeSwitch magnetic beads (commercial, + charge)	ChargeSwitch binding buffer, pH.5		ChargeSwitch wash solution AP001 + Tween20 pH 7; silicone oil		Bacteria	Cultured	dPCR		[147]
Glass beads	TEOS or APTES or GO	Acetate pH5	Tris-HCl pH7	Tris-HCl pH 9	200 µL	Bacteria + virus	Toilet seat	qPCR	0.007 CFU/cm^2^	[148]
Membrane Polyvinylidene Fluoride (PVDF)	Amine-functionalized diatomaceous earth	Dimethyl pimelimidate dihydrochloride (DMP)	PBS	Elution buffer	100 µL	Bacteria	Lab cultured	PCR qPCR		[149]
SOI wafer	APTES	Dimethyl adipimidate (DMA), 25 mg/mL	PBS	NaHCO3 10 mM, pH 10.6	50 µL	Methylated DNA	Blood, urine	PCR		[150]
Capillary	Poly-diallyl dimethylammonium chloride (PDDA)	None (thermal lysate)	no wash	No elution, in capillary amplification	-	Bacteria	Lab cultured	qPCR	10 ng/µL	[151]
MB	Chitosan	Tris, 10 mM; Triton X-100, 0.1% *v*/*v*, pH 8.5	Tris, 10 mM; Triton X-100, 0.1% *v*/*v*, pH 8.5	On-bead amplification	Virus	Whole blood	PCR	5 copes/µg of particles		
Membrane	Tertiary amine	None		Direct amplification		Bacteria	LAMP			

#### 3.1.2. Fluidics

##### Liquid Handling

**Figure 4:**
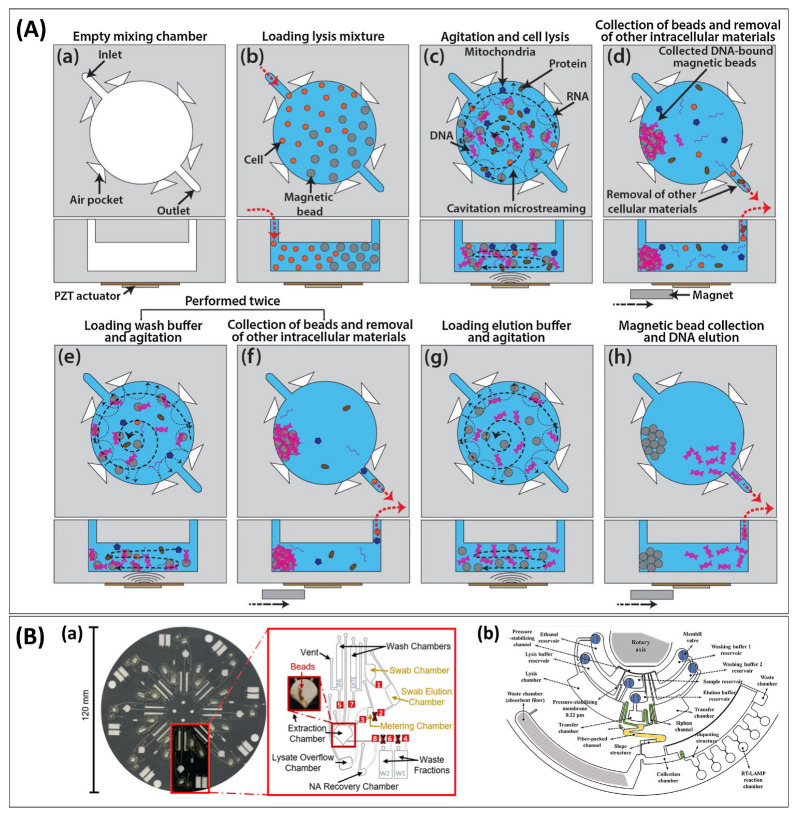
Translating the workflow. (**A**) Cavitation streaming device for lysis and mixing of reagents, showing the top view (**top** row) and side view (**bottom** row) of the microchamber for each processing step: (**a**) Cleaning of microchamber and microchannels. (**b**) Loading of magnetic beads in ethanol, followed by cells, lysis buffer, and proteinase K. (**c**) Agitation through excitation at resonance frequency; DNA binds to beads. (**d**) Suspended beads are collected into a loose aggregate just before the PZT transducer is turned off before yielding a compact aggregate at the edge of the chamber using a permanent magnet. (**e**) First wash. (**f**) Beads are collected, and waste is removed. (**e**,**f**) are repeated with a second washing buffer. (**g**) Elution under agitation. (**h**) Eluent is collected while beads are retained. Reprinted from [97] with permission from Elsevier. (**B**) Schematic representation of centrifugal force-driven microfluidic discs integrating the conventional NA sample preparation method with multi-step and -buffers for NAAT: (**a**) A multiplexed microfluidic system for dynamic SPE constructed with ten chambers (including waste fractions) pre-loaded with three different buffers. Laser-actuated valve openings and closures are indicated in red boxes and crosses, respectively, and channels with solid and dashed lines are cut into top and bottom layers, respectively. Reprinted with permission from [45]. Copyright 2021 American Chemical Society. (**b**) A disc platform with membrane-resistance valves for a sample-in-answer-out detection of avian influenza viruses built with six buffer reservoirs with different solutions, a fibre-packed channel, and seven chambers (four transfer chambers with siphon channels, and waste chambers). Reprinted from [115] with permission from Elsevier.

The loading–wash–elution workflow in SPE requires several liquid handling steps. In the laboratory, these can be conducted manually or using robots. When translating the assays to fluidic cartridges for PONT, however, new solutions need to be found to execute the workflow. For example, a laser-machined polycarbonate microfluidic chip with lysis facilitated by cavitation-microstreaming yielded comparable purity and an average concentration of mammalian DNA with a commercial extraction kit (Figure 4A). The entire procedure involved at least 20 sub-handling steps, including 17 steps for washing with ethanol and GuSCN [97]. While the chip facilitated agitation through activation of the Piezo (PZT) actuator, all liquid handling was conducted manually using pipettes. A purpose-developed pipetting cartridge was developed for the sample preparation in a fully automated sample-in-answer-out assay for the detection of *Mycobacterium tuberculosis* (MTB) [48]. Its workflow followed the standard protocol (lysis–binding–two washing steps–elution), requiring at least eight handling steps and four buffers. The eluent was then combined with RPA reagent in a droplet-based system, enabling quantification through digitization. Amidst the range of micro-scale fluid handling approaches, centrifugal, or so-called lab-on-a-disc platforms, have the advantage of providing a wide range of fluidic operations without the need for more equipment than a compact motor to rotate the device [152], with an excellent review focusing on the valving operation published elsewhere [153]. Considering the multistep workflow required for NA testing, it is not surprising that centrifugal devices have become a popular platform for NA testing. For example, a centrifugal device was developed for the detection of SARS-CoV-2 from buccal swabs using LAMP for amplification [45]. The device contained eight sets of ten chambers, three pre-loaded buffers, and packed SPE columns to perform multiplexed sample preparation, as shown in Figure 4(Ba). Similarly, a centrifugal disc built with six buffer reservoirs and seven chambers was introduced for a sample-in-answer-out detection of avian influenza viruses as shown in Figure 4(Bb) [115]. 

**Figure 5:**
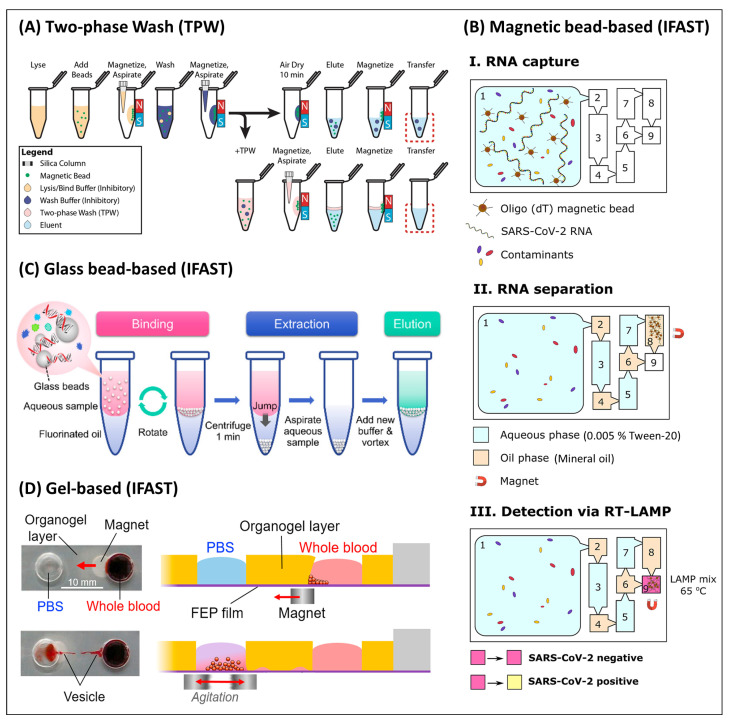
Immiscible liquids to facilitate the extraction workflow. (**A**) Schematic of TPW for MB-based NA extraction. Reprinted from [22] with permission from Springer Nature. (**B**) Workflow IFAST using four mineral oil-filled chambers to provide immiscible barriers between aqueous reagents, applied to detecting SARS-CoV-2 RNA. Reprinted from [156] with permission from Elsevier. (**C**) Workflow of in-tube IFAST using a fluorinated oil using centrifugation to relocate the glass beads from the aqueous into the oil phase. Reprinted with permission from [110]. Copyright 2019 American Chemical Society. (**D**) Photography (**left**) and schematic (**right**) using an organogel as a barrier between aqueous phases. Reprinted from [50] with permission from Elsevier.

In the translation of the NA testing workflow to a PONT format, the use of immiscible liquid interfaces has become a popular approach to transport NAs between chemical environments, loading the NAs on magnetic beads and transporting these between aqueous environments by using a magnet to drag the beads through an immiscible liquid barrier. Immiscible Phase Filtration Assisted by Surface Tension (IFAST) was introduced over a decade ago by Sur et al., demonstrating highly efficient sample preparation in a format adaptable for NA-PONT. Briefly, multiple purification steps could be executed transferring magnetic beads between aqueous environments separated by an interface made by an immiscible solvent [154,155]. Using PMPs as a solid support, the beads can be transported through the immiscible phase between chambers using a magnet, as illustrated in Figure 5B [156]. The optimal speed was tested to be approximately 1 mm/s [157,158]. The IFAST process was optimized to minimize carryover effects, caused by a thin aqueous film adhering to the particles when the beads pass through the oil, a phenomena aided by surfactants in the lysis/binding buffer that decrease the surface tension between the miscible phases [159]. For instance, an increased concentration of detergents, such as Triton X-100, SDS, and Tween-20, increases carryover across the interfacial barriers comprising liquid wax and FC-40 oil and hence should be used in moderation [159,160]. The most frequently used detergents used for lysis are Triton X-100 (0.1–1% *v*/*v*) and Tween 20 (0.005–0.1% *v*/*v*). Immiscible oils, including liquid wax, olive oil, mineral oil, silicone oil, castor oil, and FC-40, were used for IFAST system as indicated in Table 5. Mineral oil and silicon oil yielded 31.3% ± 21.5% and 41.1% ± 1.0% carryover, respectively, significantly more than FC-40 where carryover was limited to 2% owing to its relatively high density (1.85 g/mL at 25 °C). Nevertheless, mineral oil has been the most frequently used oil in recent IFAST-based PONT devices [29,41,156,161,162,163]. 

While the IFAST system is usually used with MBs, a centrifugation-assisted immiscible fluid filtration (CIFF) using glass microbeads functionalized with oligo(dT) was demonstrated (Figure 5C) [110]. CIFF utilized the differential hydrophobicity and density of elements; aqueous phase (lysis/binding buffer) > oil phase (FC-3283) > solid phase (glass beads). The difference created a vertical liquid interface where an analyte exclusion filtering system occurs. Although this approach requires centrifugation (10,000 RCF) for 1 min to transport the beads, CIFF removed approximately 99.5% of the liquid from the aqueous phase. Immiscible barriers have also been used for washing in a digital format introducing magneto fluidically enabled dPCR [147]. An overview of the use of immiscible barriers for purification is provided in Table 5.

In addition to the use immiscible liquids, gels have also been used as immiscible barriers to separate reagent conditions. An organogel-based hydrophobic purification system was presented as alternative with the potential to decrease carryover. An organogel is a gel where the fluid phase is an organic liquid, and it can be made from low-molecular-mass organogelators (LMOGs), lime 12-hydroxystearic acid (12-HAS), and an apolar liquid-like methylphenyl silicone oil, as used in Figure 5D [50]. The hardness of the organogel was optimized to allow the MBs to cross the gel while the carryover liquid was shed off due to the force restoring the deformed gel. Carryover was quantified using a dye, xylene cyanol FF, showing effective shedding of the liquid surrounding 2 mg MBs when passing through the gel. 

The use of immiscible barriers has provided an appealing approach in translating workflows from a benchtop to a fluidic platform. Most popularly applied using immiscible interfaces between liquids, IFAST has demonstrated to be highly effective for silica beads in combination with a high concentration (3–6 M) of GuSCN or GuHCl for binding. With the rise in popularity of carboxylic acid and cationic stationary phases for the use of more benign lysis reagents as discussed in Section 2.1.4 and Section 3.1.1, IFAST-based processes for cationic MBs have also been presented. Gels have also been used to provide a suitable interface that can be crossed to move between chemical environments or using electromigration, as discussed below. 

**Table 5:** Nucleic acid extraction using an immiscible phase for purification.

Immiscible Phase	Bead Surface	Mechanism	Binding	Washing	Elution	Target	Sample Matrix	Amplification	LOD	Ref.
FC-40, silicone oil, mineral oil	Silica	Chaotropic	Kit	Kit	Kit	HIV virus	Plasma	qPCR	-	[164]
FC40	silica	Chaotropic lysis	5 m GuHCl, pH 4.1 (citrate) with triton X OR Sarkoosyl OR Tween 20		Carryover study	Carryover study	Carryover study	Carryover study	Carryover study	[159]
Silicone oil	Silica	Chaotropic	Kit		Kit	HBV virus	Spiked blood	qPCR	n/r	[121]
Silicone oil	Silica	Chaotropic lysis	4 M GuSCN, 10 mM MES, 1% Triton X-100, with 1% ß-mercapto-ethanol	Oil immersed, Ethanol, 50% *v*/*v*, then water Water	None (on-bead amplification)	Virus	Nasopharyngeal swab	LAMP	1–10 copies/μL	[51]
Mineral oil	Silica	Chaotropic	GuHCl	GuHCl, 5 M	Water	Bacteria	Liquid stool (clinical)	PCR	-	[41]
Mineral oil	Silica	Chaotropic	5 M GuHCl, 0.005% TWEEN-20					LAMP-CRISPR		[163]
Mineral oil	Silica	Chaotropic	lysis buffer: Tris-HCl, lysozyme, protein kinase K, SDS, EDTA, RNase	GuHCl, 6 M	Magnesium acetate	Bacteria	Spiked milk	dRPA	10 cells	[29]
Mineral oil	Silica	Chaotropic	5 M GuHCl in 10 mM Tris-HCl 1 mM EDTA pH 8	Ethanol, 70% *v*/*v*	Water	Animal identification	Dung	qPCR, LAMP		[162]
Mineral oil	Silica	Chaotropic	3 M GuHCl			Plasmid	Cultured	dRPA	1.7e5 CFU/mL	[161]
Olive oil	Silica	Chaotropic	GuSCN, isopropanol and carrier RNA to facilitate nucleic acid precipitation		Aqueous low-salt solution	Virus	nasopharyngeal swab	RT-qPCR	12.7 ± 4.6 ng/μL	[158]
Liquid wax	Silica	Chaotropic	Alcohol			HIV	Whole blood	qPCR	1200 copies/mL (RNA)	[155]
Organogel (12-HAS)	Silica	Chaotropic	GuSCN + Triton X-100 (pH 6.8)	GITC (pH 6.8) Ethanol NaCl2 (pH 7.6)	10 mM Tris, 0.1 mM EDTA pH 8 (TE buffer)	HBV virus	Spiked blood	qPCR	5 particles	[50]
Olive oil (silicone and mineral also evaluated)	ChargeSwitch	Ionic (cationic surface)	ChargeSwitch binding buffer, pH 5							[165]
Silicone oil	ChargeSwitch	Ionic (cationic surface)	ChargeSwitch binding buffer, pH 5	ChargeSwitch wash solution AP001 + Tween20 pH 7	Direct amplification in LD Amplitaq Gold pH 8.3	Bacteria	Cultured	dPCR	Only proof of concept provided	[147]
Olive oil	ChargeSwitch	Ionic (cationic surface)	ChargeSwitch binding buffer, pH 5	ChargeSwitch washing buffer with SDS and TWEEN-20						[157]
FC-40	ZrO	Zr−O−P coordination bond and hydrogen bond	Lysis buffer: 10 mM Tris-HCl, pH 8.0, 1 mM EDTA, 0.5 mM EGTA·1% Triton X-100, 0.1% Sodium Deoxycholate, 0.1% SDS, 140 mM NaCl		10 mM PBS	Target DNA	Plasma	LAMP		[166]
Castor oil	Cellulose	Chaotropic	kit		Kit	HPV plasmids	Transport medium		10 copies/100 μL	[167]
FC-40	Silica	Chaotropic	Kit		Kit	HBV virus	Spiked plasma	dLAMP	10^4^ copies/ml	[120]
Mineral oil	(dT)coated	Chaotropic	6 M GuHCl	0.005% Tween-20	On-bead amplification	Virus	Artificial sputum	LAMP	470 copies/mL	[156]
Liquid wax olive oil	Oligo-dT PMPs	Recognition	kit	Kit	Tris-HCl	Breast cancer cells	-	RT-qPCR	-	[160]
FC-40	Biotinylated oligo	Recognition	20 mM Tris pH 7.5, 100 mM KCl, 5 mM MgCl_2_, and 0.3% Nonidet P-40/Igepal, 17U RNAseOUT™, and 2.5 μL of 100X Halt™ protease inhibitor cocktail	Diethyl pyrocarbonate in PBS	Diethyl pyrocarbonate in PBS	microRNA	Culture medium	RT-qPCR, dPCR, array		[168]

The immiscible barrier provides an effective way to remove low-molecular-weight alcohols used in washing, which is a requirement as failure to perform this can lead to inhibition of the amplification reaction [22,169]. An alternative approach to remove the alcohol is through evaporation, and this can easily be achieved on paper fluidic devices where the assay is performed in an open format. Paper fluidic devices employ the hydrophilic fibrous nature of cellulose and synthetic fibres to create fluid flow through the pores in the fibre network, which can be controlled through the introduction of hydrophobic barriers. The low cost of paper fluidic devices has led to a wide variety of applications for so-called paper analytical devices, including NA-PONT. A review dedicated to isothermal amplification can be found elsewhere [170]. For sample preparation in NA-PONT, paper has been used as a substrate simply because of this coat and fluidic control as well as the stationary phase, employing the negative charge of the cellulose to extract the NA [119]. Using its open format to aid evaporation, a paper-based microfluidic device for DNA extraction and detection of HPV from cervical specimens employed two separate washing steps with 200 µL and 100 µL of 70 and 100% ethanol, respectively, followed by air-drying by leaving on a benchtop for at least 2 min, about 20% of the total on-chip extraction time [118]. 

Considering operational aspects in a low-resource environment, a simplified kit for the extraction of cfDNA was presented in the form of a tube-based purification assay, employing three buffers: (1) 3 M GuSCN, 30% IPA, and 5% Tween 20; (2) 80% ethanol; and (3) 100% ethanol [113]. From 100 mL of plasma samples, 5.5 ng/mL of human cfDNA was recovered, which corresponds to an 84% recovery rate. An even simpler purification protocol was described using high-gradient magnetic separation (HGMS), using a relatively large volume (1.5 mL) of two different washing buffers (washing buffer 1 = 0.64 M GuSCN + 84% ethanol, washing buffer 2 = 70% ethanol) [52]. The purity was assessed using A_260_/A_280_, reaching 1.86 with a recovery rate of 10.2 ± 4.03% (sputum) and 91.2 ± 7.46% (urine). 

##### Process Integration

In the development of a PONT device, minimizing the number of reagents and processing steps is important for success. It is therefore no coincidence for many entries in Table 2, Table 3, Table 4 and Table 5 that binding takes place in lysis buffer, and these buffers have often been carefully optimized to maximize the lysis efficiency and extraction yield. At the opposite end of the workflow, the composition of the elution buffer has not received as much attention; however, it should be noted that elution can have a significant impact on the detection sensitivity because the elution volume directly affects the concentration of NAs introduced into the amplification. The optimum elution volume is a compromise of providing a sufficient volume to collect most NAs while avoiding dilution [142,171]. An automated PONT device combines extraction, amplification, and identification in a closed-type cassette, where whole blood samples are processed in a workflow finishing with an elution step in 100 µL of commercially available elution buffer [14]. Though a fully integrated and automated PONT device was developed, its sensitivity may be limited by the high elution volume. An integrated microfluidic device using cavitation microstreaming-based lysis and DNA extraction also used 100 µL elution buffer, possibly explaining its poor performance in comparison with the extraction kit [97]. Dilution during elution can be mitigated by introducing a concentration step, as demonstrated by enrichment using graphene layers in a semi-automated instrument for detecting bacteria on surfaces, outperforming the sensitivity obtained using a commercial kit even when the elution volume was 200 µL [148].

PONT assays with smaller elution volumes have demonstrated comparable performance when benchmarked with conventional extraction kits. Two devices, a multiplexed centrifugal microfluidic system for SPE from buccal swabs [45] and an IFAST-based one-step purification device for SPE from epithelial cells (MCF-7) [160], used 8 µL of TE and 8.5 µL of Tris-HCl for the elution, respectively, and the comparable qPCR results were obtained with the benchmarks. A paper-based handheld device to extract mitochondrial DNA (mtDNA) from pork samples for food adulteration required elution with 5 µL for off-chip qPCR analysis, and the results indicated an improvement in detection performance of about 1.5 times (~5 Cq values) compared with commercial DNA extraction [42]. However, the results may be influenced by high sample concentration and may not be representative for early-stage detection. 

To prevent dilution of the extract in the amplification buffer and decrease the number of reagents used, elution of the NAs into the amplification reaction mix has grown in popularity, with examples summarized in Table 6. Direct elution was performed using a filter paper-based microfluidic device, placing the paper discs into a tube containing 25 µL PCR reaction mix. Using this automated device, 8.1–21.8 ng template DNA was obtained from 0.25–1 µL blood, more than could be obtained using a commercial kit; however, the detection sensitivity was not reported [32]. Direct elution into 10 µL LAMP mix was performed to detect *Salmonella* spp. In pork meat samples by pumping the reaction mix directly into a chamber where the dried beads with extracted DNA were located [172]. This lab-on-a-chip system conducted DNA purification and amplification within 40 min for 50 CFU/reaction, while the conventional purification combined with PCR required 2–3 h to obtain the test result. An automated sample preparation device accommodating IFAST purification using silicone oil as immiscible phase and digital PCR directly eluted NAs from MBs into 65 µL of pre-loaded dPCR reaction mix [147]. The automated sample preparation was performed in 5 min, after which the beads were transferred from the device to the amplification vial for amplification. It should be noted, however, that direct elution NAAT often requires a relatively large volume of amplification mix (up to 65 µL), which increases the total cost per amplification reaction. 

Lee et al. recently introduced an even more simplified method, combining AP lysis with binding conditions known from SPRI, and named the approach abridged solid-phase extraction with alkaline poly(ethylene) glycol lysis (ASAP) [140]. The new reagents combine the lysis and binding of *E. coli* DNA in a single processing step. Owing to the benign reagent, the MBs could be introduced into the PCR mix for direct, on-bead amplification using 1.5 µg MBs per reaction. It was demonstrated that while commercial kits may be more effective in extracting the DNA from the sample, the loss and dilution during wash and elution mean that the DNA copy number present during amplification was more than an order of magnitude greater for the ASAP method using on-bead amplification. While the sensitivity could be enhanced by a 1 Cq value by introducing an ethanol wash, for most samples, this may not be justifiable with the additional reagents and handling. Elution has also been eliminated by conducting the amplification reaction that the paper substrate used for extraction/purification, for example, paper-based platforms for LAMP were recently reviewed [173]. Alternatively, LAMP reagents have been incorporated on paper as an eluent, reducing the handling of the solution and decreasing the dilution effects [174,175,176,177]. An overview of on-substrate amplification approaches is also provided in Table 6.

**Table 6:** Methods minimising dilution through elution in amplification media or elution-free systems using on-substrate amplification.

	Solid Phase	Surface	Primary Binding Principle	Elution = Amplification Mix	Volume	Target	Sample	Amplification	LOD	Reference
Elution in amplification mix	MB	Silica	Chaotrope	LAMP mix	10 μL	Bacteria	Food	LAMP	50 cells per test, or 10 cells/μL	[172]
	Paper	Whatmann FTA	Ionic interaction	LAMP mix	15.5	Lambda DNA	Saliva	LAMP	100 copies/mL	[178]
	Paper	Polyether sulfone (PES)	Chaotropic	LAMP mix	12.5	HPV 16	Cervical specimens	LAMP	1.27.10^5^ Copies	[118]
	MB	ChargeSwitch magnetic beads (commercial, + charge)	Ionic interaction	Amplitaq Gold		Bacteria	Cultured	dPCR		[147]
Elution-free	Paper	Whatmann FTA	Ionic interaction	On-paper amplification		Bacteria	Whole blood	LAMP	10 CFU/mL	[179]
	MB	Silica	Alkaline crowding	On-bead amplification		Bacteria	Artificial saliva, sweat, urine	qPCR	0.15 CFU/50 µL	[140]
	MB	Chitosan	Ionic interaction	On-bead amplification		Virus	Whole blood	PCR	5 copes/µg of particles	[180]

### 3.2. Extraction Methods without Stationary Phase

In addition to the SPE-based methods, progress has also been reported on the extraction of NAs without the use of stationary phases, and an overview is provided in Table 7. As already mentioned, MILS have been used as combined reagent for the chemical lysis and extraction of NAs without significant interference with amplification [181]. The hydrophobic MIL trihexyl(tetradecyl)phosphonium tris(hexafluoroacetylaceto)nickelate (II) ([P_6,6,6,14_^+^][Ni(hfacac)_3_^−^]) and IL ([P_6,6,6,14_^+^] [NTf_2−_]) were used for the lysis and NA extraction from plant samples. Owing to their solvating properties, the NAs were extracted into the MIL and retained with the help of a magnet allowing for the removal of the sample matrix and introduction of the amplification reagents [54]. The NAs were released from the MIL during heating, allowing for NA amplification with limited attenuation to the amplification reaction (7.9%). 

Hydrogels are swollen polymer networks that provide size selectivity in molecular transport. This selectivity has been used to allow for the transport of NAs, while preventing the transport of inhibitors, effectively allowing for extraction of the NAs from their environment. A nanoporous crosslinked PEG hydrogel, shown in Figure 3B, was used to selectively extract DNA from a complex sample matrix using the size exclusion properties of the gel (Figure 3A) [130]. PEG hydrogels have been used for different studies, such as cell migration [182], cell encapsulation [183], as well as for NAAT using digital multiple displacement amplification (dMDA) or LAMP [130,184]. For NAAT, the crosslinked nanoporous structure of the hydrogel does not attenuate amplification, but the high density of nanometer-sized pores prevents high-molecular-weight molecules, cells, and debris from entering while permitting smaller reagents to diffuse in for amplification [185]. As amplicons were confined in the gel matrix, quantification was performed by counting the fluorescent dots. The performance of the hydrogel LAMP for food safety testing outperformed the PCR and plate culture methods by shortening the sample preparation to detection time from 2–24 h to 20 min, increasing the detection sensitivity to 1 copy/µL of target bacteria from real fruit and vegetable samples [130]. Gels have also been used as a size-selective barrier, allowing for the selective migration of viral RNA into the elution chamber, from where it is collected and transferred to off-hip PCR [186]. 

**Table 7:** NA extraction approaches or PONT not employing a stationary phase.

Substrate/Method	Primary Extraction Principle	Extraction	Comment	Target	Sample Matrix	Amplification	LOD	Ref.
LLE	Preferential solubility	Various ILs and MILs	Thermal elution	White blood cells	White blood cells	qPCR	500 pg DNA from 50 µL blood	[181]
LLE	Preferential solubility	[P6,6,6,14^+^] [Ni(hfacac)3^−^] MIL, [P6,6,6,14^+^] [Co(hfacac)3^−^]^+^ Tris	Thermal elution	Plant	Plant	qPCR	311.8 ng of *A. thaliana* DNA per mg sample	[54]
Hydrogel	No binding, just physical exclusion of debris		In-gel amplification	Bacteria	Artificially infected fruits and vegetables	dLAMP	Single cell	[130]
Polyacrylamide gel	Electrophoresis	Free flow electrophoresis, followed by lysis	DNA migrates through gel after lysis	Phage	Culture medium	qPCR	1 PFU/mL or 0.02 copies/µL	[186]
ITP	Electromigration	LE 50 mM Tris HCl pH 8.2 TE 50 mM Tris HEPES pH 7.8		Lambda DNA	Blood	PCR	10 cells	[187]
ITP	Electromigration	LE 100 mM of Tris-HCl TE 100 mM Tris and 100 mM of HEPES	Hydrogel as immiscible interface	Cell-free DNA	Plasma	PCR		[188]
ITP	Electromigration	LE: 200 mM HCl with 400 mM Bistris as the LE solution TE: 10 mM Tricine with 20 mM Bistris	Paper to generate EOF counterflow, not for binding	Morpholino NA probes	-	Amplification-free detection	5 pM after 10 min	[189]
ITP	Electromigration	LE 250 mM HCl and 375 mM Tris pH 7.8; TE 25 mM serine and 25 mM Tris pH 8.7		Virus	Whole blood (spiked DNA)	RPA	1000 copies/mL	[190]
ITP	Electromigration	LE Tris HCl MgCl_2_, PEG1450, PVP, Triton X-100, and tetramethyl ammonium chloride pH 8.1 TE β-alanine, Tris, PVP, Triton X-100 pH 8.9–9.1	Paper as carrier, also focuses RPA reagents	Synthetic viral DNA	Whole blood	RPA	10^4^ copies/mL	[191]

Isotachophoresis (ITP) is an electrophoretic separation and preconcentration technique that leverages a heterogeneous buffer system to generate electric field gradients for the simultaneous focusing and separation of ionic species based on their effective electrophoretic mobilities. ITP-based purification was demonstrated for extracting genomic DNA from whole blood lysate (~10 nL on a microfluidic device, concentrating the NA in a sharp zone away from proteins and other potential interferences, allowing for the detection of spiked lambda DNA equivalent to 25 white blood cells) [187]. ITP was also used to concentrate cfDNA spiked into plasma using a microfluidic device containing removable agarose gel plates. These plates were used to create semi-permeable barriers between chambers and could be removed to collect the isotachophoretically focused DNA in that plate and transfer it to qPCR. Here, elution-free amplification was enabled by the melting of the agarose gel during the first amplification cycle, freeing the DNA [188].

ITP has also been used on paper microfluidic devices. Using the negative charge to generate an electro-osmotic flow rather than for binding, a paper fluidic device was used for processing a 200 μL sample in approximately 6 min, resulting in a 20,000-fold increase in NA concentration [189]. This device was used for amplification-free detection of nucleic acids, with a limit of detection (LoD) of 5 pM in 10 min. An acrylic cartridge was developed around a paper fluidic device for ITP of NAs from whole blood. The paper strip was integrated with a plasma separation membrane to yield plasma from whole blood, and on-paper proteolytic digestion of endogenous plasma proteins was conducted using immobilized proteinase K prior to NA extraction by ITP. After ITP, the focused band was cut out of the paper substrate using scissors, and a centrifuge was used to separate the aqueous fraction containing the NAs from the paper, after which this aqueous aliquot was transferred to the RPA amplification tube [190]. Paper-based ITP was applied to blood and plasma samples and combined with on-paper RPA, as the RPA reagents were focused with the NAs. Relatively large (20 μL of serum or 50 μL of whole blood) samples were processed without user intervention between sample loading and detection [191].

## 4. Evaluation of Sample Preparation Method for NA-PONT Assays Based on the REASSURED Criteria

The WHO’s REASSURED criteria allow for the assessment of suitability of a device/method for PONT, including Real-time connectivity, Environmentally friendly, Ease of collection, Affordable, Sensitive, Specific, User-friendly, Rapid and Robust, Equipment-free, and Deliverable to end-users. In the context of sample preparation (lysis and extraction) for NA testing, the analysis focused on the criteria Environmentally friendly, Affordable, User-friendly, Rapid, Equipment-free, and Deliverable to end-users, as other criteria are strongly dependent on instrumental, amplification, and detection aspects beyond the scope of this work. Table 8 provides an assessment of a selected number of lysis/extraction approaches reviewed in this article, with red indicating significant challenges/incompatibility, green indicating good agreement with the selected REASURED criteria, and orange indicating where there are good and poor aspects. Entry 1 presents a centrifugal platform for on-chip SPE of NAs from a lysate from buccal swabs, using guanidinium chemistry for lysis and binding [45]. Entry 2 employs an immiscible interface, organogel, to physically separate the different chemical environments for lysis, wash, and elution during the SPE of NAs on magnetic beads; transport between the environments is realized using a magnet and guanidinium chemistry is used for lysis, binding, and wash [50]. Entry 3 is a self-powered NA extraction system for an AL lysate, where the fluid handling is automated and powered by the pressure generated by a (manually activated) syringe [145]. Entry 4 employs an alkaline lysis protocol in the presence of a crowding agent to aid lysis and binding, allowing for on-bead amplification without the need for a washing step; however, the proof of concept is demonstrated in polymer test tubes and requires manual handling [140]. Entry 5 is a paper-based fluidic device that serves as a carrier for aqueous reagents that focus the NAs isolated from a detergent/enzyme-based lysis and RPA reagents into a single zone for on-paper amplification [191]. 

Environmental sustainability of the reagents used is an emerging consideration, lowering the appeal of the traditionally popular chaotropic lysis/binding using guanidium salts owing to their acute toxicity upon direct contact and their chronic toxicity to the environment, in addition to their non-sustainable production [9]. As mentioned above, guanidium salts have the advantage of providing fast lysis and facilitating binding to anionic stationary phases, which has made them attractive based on the desire to minimize reagents. The first two entries in Table 8 both employ guanidinium-based chemistry for lysis and binding and are hence marked red based on environmental concerns. Entries 3–5 have employed more benign reagents for alkaline or detergent/enzymatic lysis and are marked green. In addition to the reagents, environmental sustainability also considers the disposability or reusability of devices and the amount of plastic waste generated. Entries 1–4 all use polymer devices or tubes, limiting the sustainability (even though there is potential to substitute with biopolymers). All assays in the table were completed within the hour, so there were no significant differences in speed. The deliverability to the end-user criterion focuses on the integration of processing steps to minimize the handling and skill required for testing. For the centrifugal device (1), a sophisticated instrument is required which may limit the deliverability, whereas the ASAP method requires manual liquid handling which requires a level of training; hence, these were assessed as orange. The IFAST and paper-based systems require little skill, and the centrifugal and self-powered devices require minimal user interference. In terms of affordability, it is only the centrifugal device that is associated with significant instrumentation for use, and hence cost, recognizing the potential for the centrifugal devices to be mass-produced, leading high start-up costs but a low cost per test. The paper-based isotachophoresis test is very attractive based on the cost aspect as paper is an affordable substrate. While limited equipment is required—a power supply is required to drive the isotachophoretic separation—there is a precedent of electrophoretic separations being powered using batteries. Based on that consideration, the semiquantitative NA test with simultaneous isotachophoretic extraction and amplification has the best agreement with the REASSURED criteria, considering its high level of integration of processing steps, ease of operation, and low cost of the substrate; it is interesting to note that this approach does not use SPE for extraction.

**Table 8:** Compatibility of selected sample preparation approaches for NA-PONT with REASSURED criteria. Red means poor alignment, orange average alignment and green good alignment with the REASSURED criteria.

	Environment Lysis Reagents	Environment Extraction and Device	Equipment-Free	Deliverable to End-Users	User-Friendly	Affordable	Equipment-Free	Ref.
1. Centrifugal	Guanidium for lysis and binding	Plastic	Needs spinning	Relies on advanced instrumentation	All integrated/automated	Instrument expensive	Requires instrument	[45]
2. Organogel as immiscible barrier	Guanidium for lysis, binding, and wash	Plastic	Only needs magnet	Yes, low reliance on equipment and skill	Can be operated after limited training	No instrumentation, simple device	Yes, other than a magnet	[50]
3. Self-powered switch-controlled system	AL	Plastic device and manifold	Yes, powered by syringe (vacuum)	Yes, simple	Yes, easy to activate with gear and syringe	Simple device and tool	Syringe-powered	[145]
4. Abridged solid-phase extraction with AP lysis (ASAP)	AL	Plastic tubes	Just needs magnet (and pipette in current form)	Needs training for manual pipetting	Few reagents, no wash but in its current form relies on manual handling	Affordable reagents, but still needs packaging in device/instrument	Not in device	[140]
5. Paper-based ITP with on-paper RPA	Surfactant + enzyme	Electrolytes, paper	Membrane on paper	Paper devices easy to operate	Few reagents, all happens in electric field	Economic device, simple operation	Needs electrical power	[191]

## 5. Summary and Future Perspectives

Sample preparation plays a pivotal role in the outcome of chemical/biochemical assays conducted in a laboratory or at a point-of-need setting. Sample preparation approaches developed for laboratory-based nucleic acid amplification testing (NAAT) often entail lengthy protocols and technical complexities, rendering them unsuitable for point-of-need testing (PONT). Microfluidic technologies have emerged as appealing avenues for automating workflows originally designed for benchtop assays. Concurrent with this technological change, a growing trend towards rationalising reagent usage is motivated by the desire to reduce the number of different reagents to aid process and device simplicity, the desire to minimize reagent volume to save cost, and the desire to minimize or eliminate the use of hazardous reagents to facilitate disposal.

Despite encouraging results in the lysis of mammalian cells, non-chemical microfluidic lysis approaches including acoustic, piezoelectric, thermal, and electrical lysis are not as effective in the lysis of smaller and tougher microbial targets as chemical approaches. Consequently, chemical lysis has been most popular for NAAT, and a review of advances in lysis using detergents, enzymes, alkalinity, and chaotropic reagents indicates that combinations of multiple lytic agents often lead to increased lysis efficiency. The quest for reagents with decreased potential for attenuating the amplification reaction has led to viable alternatives to chaotropic agents. For example, AP lysis has enabled direct amplification from the lysate, minimising post-processing to a simple dilution to reduce the alkalinity. Solid-phase extraction, where NAs are bound to a solid support before elution into a purified aliquot for amplification, are popular, because an elution volume smaller than the sample volume can aid in concentration enhancement. 

Chaotropic agents can also facilitate the binding of the NA to the stationary phase during SPE. While popular in the direct translation of benchtop kits to PONT settings, concerns around the use of chaotropic agents have led to a trend away from chaotropic reagents with traditional silanol-based stationary phases towards alternate binding chemistries including the use of crowding effects and ionic binding on anionic and cationic stationary phases. The changes in binding chemistry have been accompanied with changes in the composition of binding, washing and elution buffers, for the cationic stationary phases where binding and release are facilitated by changes in pH.

In recognition of a loss in NAs transferring from the solid support to the amplification environment, dual-purpose buffers have been developed that allow for elution in amplification buffer. Direct, on-bead amplification has also been presented to eliminate elution as the stationary phase remains present during amplification.

The trends in NA-PONT are an increasing differentiation from standardized approaches developed for laboratory-based NAAT. In lysis, chemical approaches based on alkaline conditions or detergent/enzymatic approaches are expected to dominate because of the appeal of speed, efficiency, and the little environmental concern; new tailored mechanical lysis approaches are anticipated to be capable of quickly and effectively lysing microbial cells. In extraction, the use of chaotropic reagents is expected to further decrease because their manufacture is not sustainable; their use complicates the workflow and residues cannot be disposed with general waste. Several promising benign substitutes have been reported and are anticipated to further grow in popularity, as minimising processing steps and reagents will enhance affordability, user friendliness, and speed, while reducing equipment needs. In elution, the use of dual-purpose elution/amplification reagents as well as an increase in adoption of on-bead amplification are expected to boost sensitivity by preventing losses during elution. The workflow has been translated into a broad range of fluidic formats, with centrifugal devices dominating the field with integrated, automated processes; however, the sophisticated devices and instrumentation may cause challenges for implementation in a low-resource setting. More aligned with the WHO’s RE-EMERGED criteria are assays where immiscible barriers are used, allowing for the progression between processing steps of NAs immobilized on magnetic beads simply by dragging a magnet. Very well suited for PONT are the paper-based platforms, when the extraction and purification can be controlled using an electric field without the need for advanced fluidic control. In general, following trends in chemistry, fluidic design, and device manufacture, a growth in NA-PONT devices for disease surveillance/management is expected, meeting the growing demand for point-of-need tests (PONTs) for human and animal/plant health.

## Figures and Tables

**Table 1:** Chemical lysis approaches with potential for NA-PONT.

Main Lysis Method	Secondary Lysis Method	Reagents	Time/Temp (min or h/°C)	Target	Sample Matrix	Amplification	Lysis Efficiency	Yield Recovery Rate LOD	Ref.
Detergent	BSA	0.3% IGEPAL CA-630 0.1% BSA	5 min/on ice	Mammalian	N/A	RT-qPCR	N/A	N/A N/A 10 cells	[26]
	Ethanol	0.008% *Q. saponaria* 5% (*w*/*v*) NaCl 5% (*v*/*v*) ethanol	48 h/55 °C	Yeast	N/A	N/A	99.0%	N/A N/A N/A	[27]
	Enzymatic	Lysozyme Proteinase + SDS	1. 1 h/45 °C 2. 5 h/50 °C	Soil microbiome	Soil	N/A	N/A	~24 µg/g N/A N/A	[28]
	Enzymatic	10 mg/mL Lysozyme 20 ng/mL Proteinase K 0.1% SDS 1 mM EDTA 10 mM Tris-HCl 1 µL RNase	10 min/N/A	Bacteria	Milk (Spiked)	dRPA	N/A	~20 ng/µL from 10 cells N/A 10 cells	[29]
	Enzymatic	0.5% SDS 1 mg/mL Proteinase K 10 mM Dithiothreitol	15 min/65 °C	Virus	Serum (Spiked)	RT-RPA	N/A	N/A N/A 500 copies/mL	[30]
	Enzymatic	10 mM Tris-HCl (pH8) 10 mM EDTA 1% SDS 10% Triton X-100 Proteinase K DMS	20 min/56 °C	Virus	Clinical	RPA	N/A	N/A 95% 10 copies	[31]
Alkaline	N/A	1.10 mM NaOH 2. 1 mM HCl	5 min/N/A	Mammalian	Blood buccal swabs, saliva, cigarette butts	qPCR	N/A	21.8 ng/µL N/A N/A	[32]
		0.5 M NaOH 10 mM Na_2_EDTA (pH 8)	1 min/N/A	Plant	Plant	RT-RPA	N/A	N/A N/A 20 copies	[33]
	Surfactant	0.2 M NaOH 1% SDS (dried)	N/A	Bacteria	Aerosol (Spiked)	qPCR	N/A	N/A 10% 10^1^ CFU	[34]
	PEG	60% PEG200 20 mM KOH (pH 13.3–13.5)	15 min/RT	Human Animal Bacteria Plant	Raw samples	PCR	N/A	N/A N/A 10 pg	[35]
		1.25% PEG 200 10% PEG 8000 5% (*v*/*v*) NaOH	1. 3 min/RT 2. 10 min/70 °C	Virus	Whole blood (Spiked)	LAMP	100%	N/A N/A 10^2^ PFU/mL	[36]
		6% PEG 200 0.08% NaOH	3 min/RT	Fungal	Strawberry (Spiked)	RPA	N/A	N/A N/A 100 fg	[37]
		6% PEG 200 0.08% NaOH	3 min/RT	Oomycete	Leaf	RPA	N/A	N/A N/A 500 fg	[38]
		60% PEG 400 100 mM KOH	N/A	Human	Whole blood	PCR	N/A	N/A N/A N/A	[39]
		50 g/L PEG 4600 20 mM KOH (pH 13.5)	2 min/N/A	Fungal (mycelium)	Plant	LAMP	N/A	N/A N/A 19.9 pg/µL	[40]
Chaotropic	N/A	5 M GuHCl	5 min/RT	Bacteria	Liquid stool (clinical)	PCR	50% (G−) 60% (G+)	Ave 109.5 ng/µL (3-chamber) Ave 59.3 ng/µL (5-chamber) ~60% N/A	[41]
		4 M GUSCN 20 mM Tris-HCl 1 mM DTT pH 7.7	N/A	Animal	Mixed meat	qPCR	N/A	N/A N/A 0.1%	[42]
	Enzymatic	GuHCl, Proteinase K	30 min/56 °C	Bacteria	Human saliva	PCR	N/A	157.2–165 ng/µL 7.86–8.25 µg N/A	[43]
		Proteinase K GUSCN	10 min/56 °C	Bacteria	Human urine Milk	qPCR	N/A	N/A N/A 5 CFU/10 mL	[44]
		6 M GuHCl Proteinase K pH 6.1	10 min/56 °C	Virus	Buccal swab (spiked)	LAMP	N/A	N/A N/A N/A	[45]
	Detergent	6 M GuHCl 2% Triton X-100 13 mM EDTA 10 mM NaCl 51 mM Tris (pH 5.5)	5 min/RT	Virus	Serum (Spiked)	RT-PCR	N/A	1.3–2.0 µg/100 µL N/A N/A	[2]
		AMP (Melittin, Bombolitin III, MSI-78, or MSI-594)	5 min/RT	Bacteria	N/A	qLAMP	100%	N/A N/A N/A	[46]
		5 M GuSCN 100 mM EDTA 0.5% (*v*/*v*) Sarkosyl	5–10 min/N/A	Bacteria	N/A	N/A	N/A	N/A N/A N/A	[47]
		6 M GuHCl 2% Triton X-100 13 mM EDTA 10 mM NaCl 51 mM Tris pH 5.5	5 min/RT	Bacteria	Serum Saliva (Spiked)	dRPA	N/A	15–35 ng/µL 89.4%, 79.6% (saliva, serum) 1.1 × 10^8^ copies/μL	[48]
		1.5 M GuHCl 50 mM Tris [pH 8] 100 mM NaCl 5 mM EDTA 1% Tween-20	10 s/RT	Fish	Blood (Fish)	PCR	N/A	N/A N/A 10^4^ cells	[49]
		4.8% GuSCN 5% Triton X-100 (pH 6.8)	N/A	Virus	Spiked blood	PCR	N/A	N/A N/A 5 particles	[50]
		4 M GUSCN 1% Triton X-100 1% ß-mercaptoethanol 10 mM 2-Ethanesulfonic acid	5 min/RT	Virus	Nasopharyngeal swab	LAMP	N/A	N/A N/A 1–10 copies/µL	[51]
		4 M GuSCN 10 mM Tris-HCl (pH 8) 1 mM EDTA (pH 8) 0.5% Triton X-100 300 µL Isopropanol 3 µL ß-mercaptoethanol 5.6 µg poly-A carrier RNA (For RNA)	3 min/RT	Synthetic DNA	Synthetic sputum (Spiked) Residual urine sample	qPCR	N/A	N/A 10.2 ± 4.03%, 91.2 ± 7.46% (sputum, urine) N/A	[52]
		0.1 M Tris-HCl (pH8.0) 10 mM EDTA 1% SDS 10% Triton X-100 Proteinase K DNase I (RNA)	10 min/RT (RNA) 20 min/56 °C (DNA)	Mammalian Bacteria	N/A	RT-qPCR qPCR	N/A	~100 ng/µL N/A 10^3^ CUF/mL, 10^1^ cells/mL (DNA, RNA)	[53]
MIL	Peptide Enzymatic Detergent	6 µL [P_6,6,6,14_^+^] [Ni(HfAcAc)_3_^−^]	1 h/N/A	Plant	Plant	qPCR	N/A	~8 ng N/A N/A	[54]

## Abbreviations

12-HAS	12-Hydroxystearic Acid
ADE	Amine-Functionalized Diatomaceous Earth
AMPs	Antimicrobial Peptides
AL	Alkaline Lysis
AP	Alkaline polyethylene glycol-based lysis method
APTES	3-Aminopropyltriethoxysilane
ASAP	Abridged solid-phase extraction with alkaline Poly(ethylene) glycol lysis
BSA	Bovine Serum Albumin
BAW	Bulk Acoustic Wave
CAI	Centrifugation-Assisted Immiscible Fluid Filtration
cdPCR	Chamber-Based Digital PCR
cfDNA	Cell-Free DNA
CFU	Colony-Forming Unit
CMC	Critical Micellar Concentration
CMV	Cucumber Mosaic Virus
CIFF	Centrifugation-Assisted Immiscible Fluid Filtration
CRISPR	Clustered Regularly Interspaced Short Palindromic Repeats
CTAB	Cetyltrimethylammonium Bromide
cSPE	Conventional Solid-Phase Extraction
DMP	Dimethyl Pimelimidate
DNA	Deoxyribonucleic Acid
dNTP	Deoxynucleotide Triphosphate
dMDA	Digital Multiple Displacement Amplification
dPCR	Digital Polymerase Chain Reaction
DI	Deionized
DMS	Dimethyl Suberimidate
DMA	Dimethyl Adipimidate
DTT	Dithiothereitol
*E. coli*	*Escherichia coli*
EDTA	Ethylenediaminetetraacetic Acid
EL	Electrical Lysis
FC-40	Fluorinert FC-40
Fe_3_O_4_	Iron (II, III) oxide
fg	Femtogram
gDNA	Genomic DNA
GFP	Green Fluorescent Protein
GUSCN	Guanidinium Thiocyanate
Gu	Guanidine
GuHCl	Guanidine Hydrochloride
HAdV	Human Adenovirus
HBV	Hepatitis B Virus
HCl	Hydrochloric acid
HCV	Hepatitis C Virus
HCN	Hydrogen Cyanide
HEPES	4-(2-hydroxyethyl)-1-piperazineethanesulfonic acid
HGMS	High-Gradient Magnetic Separation
HI	Homobifuctional Imidoester
HPV	Human Papillomavirus
hrs	Hours
IA	Immunoassay
ICP	Ion Concentration Polarisation
ISM	Ion-Selective Membrane
IL	Ionic Liquid
ITP	Isotachophoresis
IPA	Isopropyl Alcohol
KCl	Potassium Chloride
IFAST	Immiscible Phase Filtration Assisted by Surface Tension
kb	kilo basepair
LAMP	Loop-mediated Isothermal Amplification
LLE	Liquid–Liquid Extraction
LMOGs	Low-Molecular-Mass Organogelators
LOD	Limit of Detection
MB	Magnetic Bead
MB-SPE	Magnetic Bead Solid-Phase Extraction
MES	2-ethanesulfonic Acid
mL	Millilitre
MILs	Magnetic Ionic Liquids
Min	minutes
MSIs	Magainin analogues (synthetic antimicrobial peptides)
MTB	Mycobacterium tuberculosis
mtDNA	Mitochondrial DNA
NA	Nucleic Acid
NAAT	Nucleic Acid Amplification Test
NA-PONT	Nucleic Acid Pont-Of-Need Testing
NA-SPE	Nucleic Acid Solid-Phase Extraction
NaCl	Sodium Chloride
NaHCO_3_	Sodium Hydrogencarbonate
NaOH	Sodium Hydroxide
-NH_2_	Amine group
PCR	Polymerase Chain Reaction
PDA	Polydopamine
PDMS	Polydimethylsiloxane
PEG	Polyethylene Glycol
pg	Picogram
PMPs	Paramagnetic Particles
PONT	Point-of-Need Testing
*Q. saponaria*	*Quillaja saponaria*
qPCR	Quantitative Polymerase Chain Reaction
RNA	Ribonucleic Acid
RBCs	Red Blood Cells
RT	Reverse Transcription
RT-RPA	Reverse Transcription Recombinase Polymerase Amplification
RPA	Recombinase Polymerase Amplification
SARS-CoV-2	Severe Acute Respiratory Syndrome Coronavirus 2
SAW	Surface Acoustic Wave
SDS	Sodium Dodecyl Sulfate
sec	Seconds
SIO^−^	Silanol
SPE	Solid-Phase Extraction
SPRI	Solid-Phase Reversible Immobilization
SSNES	Self-powered Switch-Controlled NA Extraction System
STT	Sodium dodecyl sulphate, Tween 20, and Triton X-100
TE	Tris-EDTA
TPW	Two-Phase Wash
µL	Microlitre

## References

[1] Ke R., Sanche S., Romero-Severson E., Hengartner N. (2020). Fast spread of COVID-19 in Europe and the US suggests the necessity of early, strong and comprehensive interventions. medRxiv.

[2] Ali Z., Wang J., Mou X., Tang Y., Li T., Liang W., Shah M.A.A., Ahmad R., Li Z., He N. (2017). Integration of Nucleic Acid Extraction Protocol with Automated Extractor for Multiplex Viral Detection. J. Nanosci. Nanotechnol..

[3] Sohrabi H., Majidi M.R., Fakhraei M., Jahanban-Esfahlan A., Hejazi M., Oroojalian F., Baradaran B., Tohidast M., Guardia M.d.l., Mokhtarzadeh A. (2022). Lateral flow assays (LFA) for detection of pathogenic bacteria: A small point-of-care platform for diagnosis of human infectious diseases. Talanta.

[4] Ince B., Sezgintürk M.K. (2022). Lateral flow assays for viruses diagnosis: Up-to-date technology and future prospects. TrAC Trends Anal. Chem..

[5] Liu W., Lee L.P. (2022). Toward Rapid and Accurate Molecular Diagnostics at Home. Adv. Mater..

[6] Boonbanjong P., Treerattrakoon K., Waiwinya W., Pitikultham P., Japrung D. (2022). Isothermal Amplification Technology for Disease Diagnosis. Biosensors.

[7] Peeling R.W., Holmes K.K., Mabey D., Ronald A. (2006). Rapid tests for sexually transmitted infections (STIs): The way forward. Sex. Transm. Infect..

[8] Manz A., Graber N., Widmer H.M. (1990). Miniaturized total chemical analysis systems: A novel concept for chemical sensing. Sens. Actuators B Chem..

[9] Otoo J.A., Schlappi T.S. (2022). REASSURED Multiplex Diagnostics: A Critical Review and Forecast. Biosensors.

[10] Lau H.Y., Botella J.R. (2017). Advanced DNA-Based Point-of-Care Diagnostic Methods for Plant Diseases Detection. Front. Plant Sci..

[11] Song Q., Sun X., Dai Z., Gao Y., Gong X., Zhou B., Wu J., Wen W. (2021). Point-of-care testing detection methods for COVID-19. Lab. A Chip..

[12] Emaus M.N., Varona M., Eitzmann D.R., Hsieh S.-A., Zeger V.R., Anderson J.L. (2020). Nucleic acid extraction: Fundamentals of sample preparation methodologies, current advancements, and future endeavors. TrAC Trends Anal. Chem..

[13] Seo M.-J., Yoo J.-C. (2020). Fully Automated Lab-On-A-Disc Platform for Loop-Mediated Isothermal Amplification Using Micro-Carbon-Activated Cell Lysis. Sensors.

[14] Dong T., Ma X., Sheng N., Qi X., Chu Y., Song Q., Zou B., Zhou G. (2021). Point-of-care DNA testing by automatically and sequentially performing extraction, amplification and identification in a closed-type cassette. Sens. Actuators B Chem..

[15] Wang S., Zhu Y., Yang Y., Li J., Hoffmann M.R. (2020). Electrochemical cell lysis of gram-positive and gram-negative bacteria: DNA extraction from environmental water samples. Electrochim. Acta.

[16] Li M., Luan Z., Liu Y., Yang C., Wang Y., Ma C., Shi C. (2021). Ultrafast bacterial cell lysis using a handheld corona treater and loop-mediated isothermal amplification for rapid detection of foodborne pathogens. Food Control.

[17] Xin Y., Xie J., Nan B., Tang C., Xiao Y., Wu Q., Lin Y., Zhang X., Shen H. (2021). Freeze-Thaw Pretreatment Can Improve Efficiency of Bacterial DNA Extraction From Meconium. Front. Microbiol..

[18] Wang W., Chen Y., Farooq U., Xuan W., Jin H., Dong S., Luo J. (2017). Ultrafast chemical-free cell lysis by high speed stream collision induced by surface acoustic waves. Appl. Phys. Lett..

[19] Deraney R.N., Schneider L., Tripathi A. (2020). Synergistic use of electroosmotic flow and magnetic forces for nucleic acid extraction. Analyst.

[20] Nan L., Jiang Z., Wei X. (2014). Emerging microfluidic devices for cell lysis: A review. Lab. A Chip..

[21] Shehadul Islam M., Aryasomayajula A., Selvaganapathy P.R. (2017). A Review on Macroscale and Microscale Cell Lysis Methods. Micromachines.

[22] Jue E., Witters D., Ismagilov R.F. (2020). Two-phase wash to solve the ubiquitous contaminant-carryover problem in commercial nucleic-acid extraction kits. Sci. Rep..

[23] Schrader C., Schielke A., Ellerbroek L., Johne R. (2012). PCR inhibitors—Occurrence, properties and removal. J. Appl. Microbiol..

[24] Paul R., Ostermann E., Wei Q. (2020). Advances in point-of-care nucleic acid extraction technologies for rapid diagnosis of human and plant diseases. Biosens. Bioelectron..

[25] Bolsover S.R., Shephard E.A., White H.A., Hyams J.S. (2011). Cell Biology: A Short Course.

[26] Viet-Phuong Le A., Huang D., Blick T., Thompson E.W., Dobrovic A. (2015). An optimised direct lysis method for gene expression studies on low cell numbers. Sci. Rep..

[27] Sewlikar S., D’Souza D.H. (2017). Antimicrobial Effects of Quillaja saponaria Extract Against *Escherichia coli* O157:H7 and the Emerging Non-O157 Shiga Toxin-Producing *E. coli*. J. Food Sci..

[28] Sakai Y. (2021). Improvements in Extraction Methods of High-molecular-weight DNA from Soils by Modifying Cell Lysis Conditions and Reducing Adsorption of DNA onto Soil Particles. Microbes Environ..

[29] Yin J., Zou Z., Hu Z., Zhang S., Zhang F., Wang B., Lv S., Mu Y. (2020). A “sample-in-multiplex-digital-answer-out” chip for fast detection of pathogens. Lab. A Chip..

[30] Bender A.T., Sullivan B.P., Zhang J.Y., Juergens D.C., Lillis L., Boyle D.S., Posner J.D. (2021). HIV detection from human serum with paper-based isotachophoretic RNA extraction and reverse transcription recombinase polymerase amplification. Analyst.

[31] Jin C.E., Lee T.Y., Koo B., Sung H., Kim S.-H., Shin Y. (2018). Rapid virus diagnostic system using bio-optical sensor and microfluidic sample processing. Sens. Actuators B Chem..

[32] Gan W., Zhuang B., Zhang P., Han J., Li C.-X., Liu P. (2014). A filter paper-based microdevice for low-cost, rapid, and automated DNA extraction and amplification from diverse sample types. Lab. A Chip..

[33] Wang X., Xie S., Chen X., Peng C., Xu X., Wei W., Ma T., Cai J., Xu J. (2020). A rapid and convenient method for on-site detection of MON863 maize through real-time fluorescence recombinase polymerase amplification. Food Chem..

[34] Seok Y., Lee J., Kim M.-G. (2021). Paper-Based Airborne Bacteria Collection and DNA Extraction Kit. Biosensors.

[35] Chomczynski P., Rymaszewski M. (2006). Alkaline polyethylene glycol-based method for direct PCR from bacteria, eukaryotic tissue samples, and whole blood. BioTechniques.

[36] Yoo H.J., Baek C., Lee M.-H., Min J. (2020). Integrated microsystems for the in situ genetic detection of dengue virus in whole blood using direct sample preparation and isothermal amplification. Analyst.

[37] Lu X., Xu H., Song W., Yang Z., Yu J., Tian Y., Jiang M., Shen D., Dou D. (2021). Rapid and simple detection of Phytophthora cactorum in strawberry using a coupled recombinase polymerase amplification–lateral flow strip assay. Phytopathol. Res..

[38] Lu X., Zheng Y., Zhang F., Yu J., Dai T., Wang R., Tian Y., Xu H., Shen D., Dou D. (2020). A Rapid, Equipment-Free Method for Detecting Phytophthora infestans in the Field Using a Lateral Flow Strip-Based Recombinase Polymerase Amplification Assay. Plant Dis..

[39] Liu X., Zhang C., Hua K., Liang J., Li H., Ma T., Zhu J., Cui Y. (2019). Direct genotyping from whole blood using alkaline polyethylene glycol. Anal. Biochem..

[40] Sillo F., Giordano L., Gonthier P. (2018). Fast and specific detection of the invasive forest pathogen Heterobasidion irregulare through a Loop-mediated isothermal AMPlification (LAMP) assay. For. Pathol..

[41] Mosley O., Melling L., Tarn M.D., Kemp C., Esfahani M.M.N., Pamme N., Shaw K.J. (2016). Sample introduction interface for on-chip nucleic acid-based analysis of *Helicobacter pylori* from stool samples. Lab. A Chip..

[42] Batule B.S., Seok Y., Kim M.-G. (2020). An innovative paper-based device for DNA extraction from processed meat products. Food Chem..

[43] Bhati A., Varghese A., Rajan G., Sridhar V., Mohan Y., Pradeep S., Babu S., Kaikkolante N., Sarma M., Arun S. (2021). An effective method for saliva stabilization and magnetic nanoparticles based DNA extraction for genomic applications. Anal. Biochem..

[44] Chen F., Kim S., Na J.-H., Han K., Lee T.Y. (2020). A single-tube sample preparation method based on a dual-electrostatic interaction strategy for molecular diagnosis of gram-negative bacteria. Microchim. Acta.

[45] Dignan L.M., Woolf M.S., Tomley C.J., Nauman A.Q., Landers J.P. (2021). Multiplexed Centrifugal Microfluidic System for Dynamic Solid-Phase Purification of Polynucleic Acids Direct from Buccal Swabs. Anal. Chem..

[46] Krõlov K., Uusna J., Grellier T., Andresen L., Jevtuševskaja J., Tulp I., Langel Ü. (2017). Implementation of antimicrobial peptides for sample preparation prior to nucleic acid amplification in point-of-care settings. Expert Rev. Mol. Diagn..

[47] Pitcher D.G., Saunders N.A., Owen R.J. (1989). Rapid extraction of bacterial genomic DNA with guanidium thiocyanate. Lett. Appl. Microbiol..

[48] Yang H., Chen Z., Cao X., Li Z., Stavrakis S., Choo J., deMello A.J., Howes P.D., He N. (2018). A sample-in-digital-answer-out system for rapid detection and quantitation of infectious pathogens in bodily fluids. Anal. Bioanal. Chem..

[49] Gui L., Li X., Lin S., Zhao Y., Lin P., Wang B., Tang R., Guo J., Zu Y., Zhou Y. (2022). Low-Cost and Rapid Method of DNA Extraction from Scaled Fish Blood and Skin Mucus. Viruses.

[50] Ohashi T., Kuyama H. (2020). Magnetic particle transport through organogel—An application to DNA extraction. Anal. Biochem..

[51] Juang D.S., Juang T.D., Dudley D.M., Newman C.M., Accola M.A., Rehrauer W.M., Friedrich T.C., O’Connor D.H., Beebe D.J. (2021). Oil immersed lossless total analysis system for integrated RNA extraction and detection of SARS-CoV-2. Nat. Commun..

[52] Pearlman S.I., Leelawong M., Richardson K.A., Adams N.M., Russ P.K., Pask M.E., Wolfe A.E., Wessely C., Haselton F.R. (2020). Low-Resource Nucleic Acid Extraction Method Enabled by High-Gradient Magnetic Separation. ACS Appl. Mater. Interfaces.

[53] Jin C.E., Lee T.Y., Koo B., Choi K.-C., Chang S., Park S.Y., Kim J.Y., Kim S.-H., Shin Y. (2017). Use of Dimethyl Pimelimidate with Microfluidic System for Nucleic Acids Extraction without Electricity. Anal. Chem..

[54] Emaus M.N., Cagliero C., Gostel M.R., Johnson G., Anderson J.L. (2022). Simple and efficient isolation of plant genomic DNA using magnetic ionic liquids. Plant Methods.

[55] Helenius A., Simons K. (1975). Solubilization of membranes by detergents. Biochim. Biophys. Acta (BBA) Rev. Biomembr..

[56] Lichtenberg D., Ahyayauch H., Goñi F.M. (2013). The mechanism of detergent solubilization of lipid bilayers. Biophys. J..

[57] Ahyayauch H., Bennouna M., Alonso A., Goñi F.M. (2010). Detergent Effects on Membranes at Subsolubilizing Concentrations: Transmembrane Lipid Motion, Bilayer Permeabilization, and Vesicle Lysis/Reassembly Are Independent Phenomena. Langmuir.

[58] Berezovski M.V., Mak T.W., Krylov S.N. (2007). Cell lysis inside the capillary facilitated by transverse diffusion of laminar flow profiles (TDLFP). Anal. Bioanal. Chem..

[59] Syn C.K., Teo W.L., Swarup S. (1999). Three-detergent method for the extraction of RNA from several bacteria. BioTechniques.

[60] Berlowska J., Dudkiewicz M., Kregiel D., Czyzowska A., Witonska I. (2015). Cell lysis induced by membrane-damaging detergent saponins from Quillaja saponaria. Enzym. Microb. Technol..

[61] Partearroyo M.A., Ostolaza H., Goñi F.M., Barberá-Guillem E. (1990). Surfactant-induced cell toxicity and cell lysis: A study using B16 melanoma cells. Biochem. Pharmacol..

[62] Jones S.A., Laskaris G., Vincent-Bonnieu S., Farajzadeh R., Rossen W.R. (2016). Effect of surfactant concentration on foam: From coreflood experiments to implicit-texture foam-model parameters. J. Ind. Eng. Chem..

[63] Pereiro I., Fomitcheva Khartchenko A., Petrini L., Kaigala G.V. (2019). Nip the bubble in the bud: A guide to avoid gas nucleation in microfluidics. Lab. A Chip..

[64] Higuchi R. (1989). Simple and rapid preparation of samples for PCR. PCR Technology.

[65] Villarreal J.V., Jungfer C., Obst U., Schwartz T. (2013). DNase I and Proteinase K eliminate DNA from injured or dead bacteria but not from living bacteria in microbial reference systems and natural drinking water biofilms for subsequent molecular biology analyses. J. Microbiol. Methods.

[66] Genoud V., Stortz M., Waisman A., Berardino B.G., Verneri P., Dansey V., Salvatori M., Remes Lenicov F., Levi V. (2021). Extraction-free protocol combining proteinase K and heat inactivation for detection of SARS-CoV-2 by RT-qPCR. PLoS ONE.

[67] Bera A., Herbert S., Jakob A., Vollmer W., Götz F. (2005). Why are pathogenic staphylococci so lysozyme resistant? The peptidoglycan O-acetyltransferase OatA is the major determinant for lysozyme resistance of Staphylococcus aureus. Mol. Microbiol..

[68] Li S., Norioka S., Sakiyama F. (1997). Purification, staphylolytic activity, and cleavage sites of α-lytic protease from Achromobacter lyticus. J. Biochem..

[69] Heiniger E.K., Buser J.R., Mireles L., Zhang X., Ladd P.D., Lutz B.R., Yager P. (2016). Comparison of point-of-care-compatible lysis methods for bacteria and viruses. J. Microbiol. Methods.

[70] Shah K.G., Roller M., Kumar S., Bennett S., Heiniger E., Looney K., Buser J., Bishop J.D., Yager P. (2023). Disposable platform for bacterial lysis and nucleic acid amplification based on a single USB-powered printed circuit board. PLoS ONE.

[71] Buser J.R., Zhang X., Byrnes S.A., Ladd P.D., Heiniger E.K., Wheeler M.D., Bishop J.D., Englund J.A., Lutz B., Weigl B.H. (2016). A disposable chemical heater and dry enzyme preparation for lysis and extraction of DNA and RNA from microorganisms. Anal. Methods.

[72] Chondrogiannis G., Réu P., Hamedi M.M. (2023). Paper-Based Bacterial Lysis Enables Sample-to-Answer Home-based DNA Testing. Adv. Mater. Technol..

[73] Birnboim H.C., Doly J. (1979). A rapid alkaline extraction procedure for screening recombinant plasmid DNA. Nucleic Acids Res..

[74] Green M.R., Sambrook J. (2016). Preparation of Plasmid DNA by Alkaline Lysis with Sodium Dodecyl Sulfate: Minipreps. Cold Spring Harb. Protoc..

[75] Jiang H., Panda S., Gekara N.O. (2019). Chapter Eighteen—Comet and micronucleus assays for analyzing DNA damage and genome integrity. Methods Enzymol..

[76] Ickenstein L.M., Sandström M.C., Mayer L.D., Edwards K. (2006). Effects of phospholipid hydrolysis on the aggregate structure in DPPC/DSPE-PEG2000 liposome preparations after gel to liquid crystalline phase transition. Biochim. Biophys. Acta Biomembr..

[77] Girish P.S., Barbuddhe S.B., Kumari A., Rawool D.B., Karabasanavar N.S., Muthukumar M., Vaithiyanathan S. (2020). Rapid detection of pork using alkaline lysis- Loop Mediated Isothermal Amplification (AL-LAMP) technique. Food Control.

[78] Zhao G., Wang J., Yao C., Xie P., Li X., Xu Z., Xian Y., Lei H., Shen X. (2022). Alkaline lysis-recombinase polymerase amplification combined with CRISPR/Cas12a assay for the ultrafast visual identification of pork in meat products. Food Chem..

[79] Gautam A., Gautam A. (2022). Isolation of Plasmid DNA by Alkaline Lysis. DNA and RNA Isolation Techniques for Non-Experts.

[80] Salvi G., De Los Rios P., Vendruscolo M. (2005). Effective interactions between chaotropic agents and proteins. Proteins Struct. Funct. Bioinform..

[81] Melzak K.A., Sherwood C.S., Turner R.F.B., Haynes C.A. (1996). Driving Forces for DNA Adsorption to Silica in Perchlorate Solutions. J. Colloid Interface Sci..

[82] Yang W. (2011). Nucleases: Diversity of structure, function and mechanism. Q. Rev. Biophys..

[83] Lee S., Kim S., Kim S. (2023). A novel paper-based lysis strip for SARS-CoV-2 RNA detection at low resource settings. Anal. Biochem..

[84] Zhang Y., Ren G., Buss J., Barry A.J., Patton G.C., Tanner N.A. (2020). Enhancing colorimetric loop-mediated isothermal amplification speed and sensitivity with guanidine chloride. BioTechniques.

[85] Cho H.-S., Choi M., Lee Y., Jeon H., Ahn B., Soundrarajan N., Hong K., Kim J.-H., Park C. (2021). High-Quality Nucleic Acid Isolation from Hard-to-Lyse Bacterial Strains Using PMAP-36, a Broad-Spectrum Antimicrobial Peptide. Int. J. Mol. Sci..

[86] Lei Z., Chen B., Koo Y.-M., MacFarlane D.R. (2017). Introduction: Ionic Liquids. Chem. Rev..

[87] Jiang K., Liu L., Liu X., Zhang X., Zhang S. (2019). Insight into the Relationship between Viscosity and Hydrogen Bond of a Series of Imidazolium Ionic Liquids: A Molecular Dynamics and Density Functional Theory Study. Ind. Eng. Chem. Res..

[88] George A., Brandt A., Tran K., Zahari S.M.S.N.S., Klein-Marcuschamer D., Sun N., Sathitsuksanoh N., Shi J., Stavila V., Parthasarathi R. (2015). Design of low-cost ionic liquids for lignocellulosic biomass pretreatment. Green Chem..

[89] Vandeventer P.E., Weigel K.M., Salazar J., Erwin B., Irvine B., Doebler R., Nadim A., Cangelosi G.A., Niemz A. (2011). Mechanical disruption of lysis-resistant bacterial cells by use of a miniature, low-power, disposable device. J. Clin. Microbiol..

[90] Lu H.-W., Sakamuri R., Kumar P., Ferguson T.M., Doebler R.W., Herrington K.D., Talbot R.P., Weigel K.M., Nguyen F.K., Cangelosi G.A. (2020). Integrated nucleic acid testing system to enable TB diagnosis in peripheral settings. Lab. A Chip..

[91] Shin D.J., Athamanolap P., Chen L., Hardick J., Lewis M., Hsieh Y.H., Rothman R.E., Gaydos C.A., Wang T.H. (2017). Mobile nucleic acid amplification testing (mobiNAAT) for Chlamydia trachomatis screening in hospital emergency department settings. Sci. Rep..

[92] Grigorov E., Kirov B., Marinov M.B., Galabov V. (2021). Review of Microfluidic Methods for Cellular Lysis. Micromachines.

[93] Danaeifar M. (2022). New horizons in developing cell lysis methods: A review. Biotechnol. Bioeng..

[94] Marentis T.C., Kusler B., Yaralioglu G.G., Liu S., Haeggström E.O., Khuri-Yakub B.T. (2005). Microfluidic sonicator for real-time disruption of eukaryotic cells and bacterial spores for DNA analysis. Ultrasound Med. Biol..

[95] Branch D.W., Vreeland E.C., McClain J.L., Murton J.K., James C.D., Achyuthan K.E. (2017). Rapid Nucleic Acid Extraction and Purification Using a Miniature Ultrasonic Technique. Micromachines.

[96] Lu H., Mutafopulos K., Heyman J.A., Spink P., Shen L., Wang C., Franke T., Weitz D.A. (2019). Rapid additive-free bacteria lysis using traveling surface acoustic waves in microfluidic channels. Lab. A Chip..

[97] Kaba A.M., Jeon H., Park A., Yi K., Baek S., Park A., Kim D. (2021). Cavitation-microstreaming-based lysis and DNA extraction using a laser-machined polycarbonate microfluidic chip. Sens. Actuators B Chem..

[98] Zevnik J., Dular M. (2022). Cavitation bubble interaction with compliant structures on a microscale: A contribution to the understanding of bacterial cell lysis by cavitation treatment. Ultrason. Sonochem..

[99] Zupanc M., Zevnik J., Filipić A., Gutierrez-Aguirre I., Ješelnik M., Košir T., Ortar J., Dular M., Petkovšek M. (2023). Inactivation of the enveloped virus phi6 with hydrodynamic cavitation. Ultrason. Sonochem..

[100] Nittala P.V.K., Hohreiter A., Rosas Linhard E., Dohn R., Mishra S., Konda A., Divan R., Guha S., Basu A. (2023). Integration of silicon chip microstructures for in-line microbial cell lysis in soft microfluidics. Lab. A Chip..

[101] Kim Y., Kim S. (2018). An electro-conductive plane heating element for rapid thermal lysis of bacterial cells. J. Microbiol. Methods.

[102] Shetty P., Ghosh D., Paul D. (2017). Thermal lysis and isothermal amplification of Mycobacterium tuberculosis H37Rv in one tube. J. Microbiol. Methods.

[103] Kim M., Wu L., Kim B., Hung D.T., Han J. (2018). Continuous and High-Throughput Electromechanical Lysis of Bacterial Pathogens Using Ion Concentration Polarization. Anal. Chem..

[104] Zhao J., Li N., Zhou X., Yu Z., Lan M., Chen S., Miao J., Li Y., Li G., Yang F. (2023). Electrolysis of Bacteria Based on Microfluidic Technology. Micromachines.

[105] Ma S., Bryson B.D., Sun C., Fortune S.M., Lu C. (2016). RNA Extraction from a Mycobacterium under Ultrahigh Electric Field Intensity in a Microfluidic Device. Anal. Chem..

[106] Islam M.S., Shahid A., Kuryllo K., Li Y., Deen M.J., Selvaganapathy P.R. (2017). Electrophoretic Concentration and Electrical Lysis of Bacteria in a Microfluidic Device Using a Nanoporous Membrane. Micromachines.

[107] Hong S., Park K.S., Weissleder R., Castro C.M., Lee H. (2017). Facile silicification of plastic surface for bioassays. Chem. Commun..

[108] Boom R., Sol C.J., Salimans M.M., Jansen C.L., Wertheim-van Dillen P.M., van der Noordaa J. (1990). Rapid and simple method for purification of nucleic acids. J. Clin. Microbiol..

[109] Xu P., Wang H., Tong R., Du Q., Zhong W. (2006). Preparation and morphology of SiO2/PMMA nanohybrids by microemulsion polymerization. Colloid Polym. Sci..

[110] Juang D.S., Berry S.M., Li C., Lang J.M., Beebe D.J. (2019). Centrifugation-Assisted Immiscible Fluid Filtration for Dual-Bioanalyte Extraction. Anal. Chem..

[111] Ngo D., Liu H., Chen Z., Kaya H., Zimudzi T.J., Gin S., Mahadevan T., Du J., Kim S.H. (2020). Hydrogen bonding interactions of H_2_O and SiOH on a boroaluminosilicate glass corroded in aqueous solution. NPJ Mater. Degrad..

[112] Wang R., Wu J., He X., Zhou P., Shen Z. (2021). A Sample-In-Answer-Out Microfluidic System for the Molecular Diagnostics of 24 HPV Genotypes Using Palm-Sized Cartridge. Micromachines.

[113] Raymond C.K., Raymond F.C., Hill K. (2020). UltraPrep is a scalable, cost-effective, bead-based method for purifying cell-free DNA. PLoS ONE.

[114] Du K., Cai H., Park M., Wall T.A., Stott M.A., Alfson K.J., Griffiths A., Carrion R., Patterson J.L., Hawkins A.R. (2017). Multiplexed efficient on-chip sample preparation and sensitive amplification-free detection of Ebola virus. Biosens. Bioelectron..

[115] Liu Q., Zhang X., Chen L., Yao Y., Ke S., Zhao W., Yang Z., Sui G. (2018). A sample-to-answer labdisc platform integrated novel membrane-resistance valves for detection of highly pathogenic avian influenza viruses. Sens. Actuators B Chem..

[116] Chen X., Cui D., Liu C., Li H., Chen J. (2007). Continuous flow microfluidic device for cell separation, cell lysis and DNA purification. Anal. Chim. Acta.

[117] Gulliksen A., Keegan H., Martin C., O’Leary J., Solli L.A., Falang I.M., Grønn P., Karlgård A., Mielnik M.M., Johansen I.-R. (2012). Towards a “Sample-In, Answer-Out” Point-of-Care Platform for Nucleic Acid Extraction and Amplification: Using an HPV E6/E7 mRNA Model System. J. Oncol..

[118] Rodriguez N.M., Wong W.S., Liu L., Dewar R., Klapperich C.M. (2016). A fully integrated paperfluidic molecular diagnostic chip for the extraction, amplification, and detection of nucleic acids from clinical samples. Lab. A Chip..

[119] Linnes J.C., Fan A., Rodriguez N.M., Lemieux B., Kong H., Klapperich C.M. (2014). Paper-based molecular diagnostic for Chlamydia trachomatis. RSC Adv..

[120] Hu F., Li J., Zhang Z., Li M., Zhao S., Li Z., Peng N. (2020). Smartphone-Based Droplet Digital LAMP Device with Rapid Nucleic Acid Isolation for Highly Sensitive Point-of-Care Detection. Anal. Chem..

[121] Hu F., Li J., Peng N., Li Z., Zhang Z., Zhao S., Duan M., Tian H., Li L., Zhang P. (2019). Rapid isolation of cfDNA from large-volume whole blood on a centrifugal microfluidic chip based on immiscible phase filtration. Analyst.

[122] Hallsworth J.E. (1998). Ethanol-induced water stress in yeast. J. Ferment. Bioeng..

[123] Page R., Scourfield E., Ficarelli M., McKellar S.W., Lee K.L., Maguire T.J.A., Bouton C., Lista M.J., Neil S.J.D., Malim M.H. (2022). Homebrew: An economical and sensitive glassmilk-based nucleic-acid extraction method for SARS-CoV-2 diagnostics. Cell Rep. Methods.

[124] Wang Z., Wang Y., Lin L., Wu T., Zhao Z., Ying B., Chang L. (2022). A finger-driven disposable micro-platform based on isothermal amplification for the application of multiplexed and point-of-care diagnosis of tuberculosis. Biosens. Bioelectron..

[125] Miller S.A., Dykes D.D., Polesky H.F. (1988). A simple salting out procedure for extracting DNA from human nucleated cells. Nucleic Acids Res..

[126] Mohsen-Nia M., Amiri H., Jazi B. (2010). Dielectric Constants of Water, Methanol, Ethanol, Butanol and Acetone: Measurement and Computational Study. J. Solut. Chem..

[127] Park J.-G., Lee S.-H., Ryu J.-S., Hong Y.-K., Kim T.-G., Busnaina A.A. (2006). Interfacial and Electrokinetic Characterization of IPA Solutions Related to Semiconductor Wafer Drying and Cleaning. J. Electrochem. Soc..

[128] Lee W.D., Gawri R., Shiba T., Ji A.-R., Stanford W.L., Kandel R.A. (2017). Simple Silica Column–Based Method to Quantify Inorganic Polyphosphates in Cartilage and Other Tissues. Cartilage.

[129] Kumar S., Kharb A., Vazirani A., Chauhan R.S., Pramanik G., Sengupta M., Ghosh S. (2022). Nucleic acid extraction from complex biofluid using toothpick-actuated over-the-counter medical-grade cotton. Bioorganic Med. Chem..

[130] Lin X., Fang M., Yi C., Jiang Y., Zhang C., Pan X., Luo Z. (2022). Functional hydrogel for fast, precise and inhibition-free point-of-care bacteria analysis in crude food samples. Biomaterials.

[131] Zhao F., Lee E.Y., Noh G.S., Shin J., Liu H., Qiao Z., Shin Y. (2019). A robust, hand-powered, instrument-free sample preparation system for point-of-care pathogen detection. Sci. Rep..

[132] Akabayov B., Akabayov S.R., Lee S.-J., Wagner G., Richardson C.C. (2013). Impact of macromolecular crowding on DNA replication. Nat. Commun..

[133] Phillip Y., Sherman E., Haran G., Schreiber G. (2009). Common crowding agents have only a small effect on protein-protein interactions. Biophys. J..

[134] Miyoshi D., Sugimoto N. (2008). Molecular crowding effects on structure and stability of DNA. Biochimie.

[135] Hawkins T.L., O’Connor-Morin T., Roy A., Santillan C. (1994). DNA purification and isolation using a solid-phase. Nucleic Acids Res..

[136] Rohland N., Reich D. (2012). Cost-effective, high-throughput DNA sequencing libraries for multiplexed target capture. Genome Res..

[137] Maghini D.G., Moss E.L., Vance S.E., Bhatt A.S. (2021). Improved high-molecular-weight DNA extraction, nanopore sequencing and metagenomic assembly from the human gut microbiome. Nat. Protoc..

[138] Stortchevoi A., Kamelamela N., Levine S.S. (2020). SPRI Beads-based Size Selection in the Range of 2–10 kb. J. Biomol. Tech..

[139] Vo A.T.E., Jedlicka J.A. (2014). Protocols for metagenomic DNA extraction and Illumina amplicon library preparation for faecal and swab samples. Mol. Ecol. Resour..

[140] Lee S.M., Nai Y.H., Doeven E.H., Balakrishnan H.K., Yuan D., Guijt R.M. (2023). Abridged solid-phase extraction with alkaline Poly(ethylene) glycol lysis (ASAP) for direct DNA amplification. Talanta.

[141] Kastania A.S., Petrou P.S., Loukas C.-M., Gogolides E. (2020). Poly-L-histidine coated microfluidic devices for bacterial DNA purification without chaotropic solutions. Biomed. Microdevices.

[142] Zhang J., Su X., Xu J., Wang J., Zeng J., Li C., Chen W., Li T., Min X., Zhang D. (2019). A point of care platform based on microfluidic chip for nucleic acid extraction in less than 1 minute. Biomicrofluidics.

[143] Zandieh M., Liu J. (2021). Spherical Nucleic Acid Mediated Functionalization of Polydopamine-Coated Nanoparticles for Selective DNA Extraction and Detection. Bioconjugate Chem..

[144] Seong H., Park J., Bae M., Shin S. (2022). Rapid and Efficient Extraction of Cell-Free DNA Using Homobifunctional Crosslinkers. Biomedicines.

[145] Han K., Yoon Y.-J., Shin Y., Park M.K. (2016). Self-powered switch-controlled nucleic acid extraction system. Lab. A Chip..

[146] Hermanson G.T., Hermanson G.T. (2013). Chapter 5—Homobifunctional Crosslinkers. Bioconjugate Techniques.

[147] Gaddes D.E., Lee P.-W., Trick A.Y., Athamanolap P., O’Keefe C.M., Puleo C., Hsieh K., Wang T.-H. (2020). Facile Coupling of Droplet Magnetofluidic-Enabled Automated Sample Preparation for Digital Nucleic Acid Amplification Testing and Analysis. Anal. Chem..

[148] Lee W.-N., Yoo H.J., Nguyen K.H., Baek C., Min J. (2019). Semi-automatic instrumentation for nucleic acid extraction and purification to quantify pathogens on surfaces. Analyst.

[149] Noh G.S., Liu H., Kim M.G., Qiao Z., Jang Y.O., Shin Y. (2020). Multi-Sample Preparation Assay for Isolation of Nucleic Acids Using Bio-Silica with Syringe Filters. Micromachines.

[150] Shin Y., Perera A.P., Wong C.C., Park M.K. (2014). Solid phase nucleic acid extraction technique in a microfluidic chip using a novel non-chaotropic agent: Dimethyl adipimidate. Lab. A Chip..

[151] Fu Y., Zhou X., Xing D. (2017). Lab-on-capillary: A rapid, simple and quantitative genetic analysis platform integrating nucleic acid extraction, amplification and detection. Lab. A Chip..

[152] O’Connell K.C., Landers J.P. (2023). Integrated membranes within centrifugal microfluidic devices: A review. Lab. A Chip..

[153] Ducrée J. (2021). Systematic review of centrifugal valving based on digital twin modeling towards highly integrated lab-on-a-disc systems. Microsyst. Nanoeng..

[154] Chen Y., Liu Y., Shi Y., Ping J., Wu J., Chen H. (2020). Magnetic particles for integrated nucleic acid purification, amplification and detection without pipetting. TrAC Trends Anal. Chem..

[155] Sur K., McFall S.M., Yeh E.T., Jangam S.R., Hayden M.A., Stroupe S.D., Kelso D.M. (2010). Immiscible Phase Nucleic Acid Purification Eliminates PCR Inhibitors with a Single Pass of Paramagnetic Particles through a Hydrophobic Liquid. J. Mol. Diagn..

[156] Rodriguez-Mateos P., Ngamsom B., Walter C., Dyer C.E., Gitaka J., Iles A., Pamme N. (2021). A lab-on-a-chip platform for integrated extraction and detection of SARS-CoV-2 RNA in resource-limited settings. Anal. Chim. Acta.

[157] Troiano D., Deraney R.N., Tripathi A. (2017). Effect of surfactants on carryover liquid volume in immiscible phase magnetic bead separation. Colloids Surf. A Physicochem. Eng. Asp..

[158] Cui F.R., Wang J., Opal S.M., Tripathi A. (2016). Isolating Influenza RNA from Clinical Samples Using Microfluidic Oil-Water Interfaces. PLoS ONE.

[159] Kistrup K., Skotte Sørensen K., Wolff A., Fougt Hansen M. (2015). Liquid carry-over in an injection moulded all-polymer chip system for immiscible phase magnetic bead-based solid-phase extraction. J. Magn. Magn. Mater..

[160] Berry S.M., Alarid E.T., Beebe D.J. (2011). One-step purification of nucleic acid for gene expression analysis via Immiscible Filtration Assisted by Surface Tension (IFAST). Lab. A Chip..

[161] Kalsi S., Valiadi M., Turner C., Sutton M., Morgan H. (2019). Sample pre-concentration on a digital microfluidic platform for rapid AMR detection in urine. Lab. A Chip..

[162] Wimbles R., Melling L.M., Cain B., Davies N., Doherty J., Johnson B., Shaw K.J. (2021). On-site genetic analysis for species identification using lab-on-a-chip. Ecol. Evol..

[163] Ngamsom B., Iles A., Kamita M., Kimani R., Wakaba P., Rodriguez-Mateos P., Mungai M., Dyer C.E., Walter C., Gitaka J. (2022). A sample-to-answer COVID-19 diagnostic device based on immiscible filtration and CRISPR-Cas12a-assisted detection. Talanta Open.

[164] Berry S.M., LaVanway A.J., Pezzi H.M., Guckenberger D.J., Anderson M.A., Loeb J.M., Beebe D.J. (2014). HIV Viral RNA Extraction in Wax Immiscible Filtration Assisted by Surface Tension (IFAST) Devices. J. Mol. Diagn..

[165] Strotman L.N., Lin G., Berry S.M., Johnson E.A., Beebe D.J. (2012). Facile and rapid DNA extraction and purification from food matrices using IFAST (immiscible filtration assisted by surface tension). Analyst.

[166] Pan W., Wang X., Ma X., Chu Y.n., Pang S., Chen Y., Guan X., Zou B., Wu Y., Zhou G. (2021). Postsynthetic Modification of the Magnetic Zirconium–Organic Framework for Efficient and Rapid Solid-Phase Extraction of DNA. ACS Appl. Mater. Interfaces.

[167] Zhang L., Deraney R.N., Tripathi A. (2015). Adsorption and isolation of nucleic acids on cellulose magnetic beads using a three-dimensional printed microfluidic chip. Biomicrofluidics.

[168] Poenitzsch Strong A.M., Berry S.M., Beebe D.J., Li J.L., Spiegelman V.S. (2018). miFAST: A novel and rapid microRNA target capture method. Mol. Carcinog.

[169] Claveau S., Sasseville M., Beauregard M. (2004). Alcohol-Mediated Error-Prone PCR. DNA Cell Biol..

[170] Reynolds J., Loeffler R.S., Leigh P.J., Lopez H.A., Yoon J.-Y. (2023). Recent Uses of Paper Microfluidics in Isothermal Nucleic Acid Amplification Tests. Biosensors.

[171] Kang J.-H., Kim Y.T., Lee K., Kim H.-M., Lee K.G., Ahn J., Lee J., Lee S.J., Kim K.-B. (2020). An electrophoretic DNA extraction device using a nanofilter for molecular diagnosis of pathogens. Nanoscale.

[172] Sun Y., Quyen T.L., Hung T.Q., Chin W.H., Wolff A., Bang D.D. (2015). A lab-on-a-chip system with integrated sample preparation and loop-mediated isothermal amplification for rapid and quantitative detection of *Salmonella* spp. in food samples. Lab. A Chip..

[173] Das D., Masetty M., Priye A. (2023). Paper-Based Loop Mediated Isothermal Amplification (LAMP) Platforms: Integrating the Versatility of Paper Microfluidics with Accuracy of Nucleic Acid Amplification Tests. Chemosensors.

[174] Kaur N., Toley B.J. (2023). Tuberculosis Diagnosis Using Isothermal Nucleic Acid Amplification in a Paper-and-Plastic Device. Methods Mol. Biol..

[175] Jawla J., Kumar R.R., Mendiratta S.K., Agarwal R.K., Singh P., Saxena V., Kumari S., Kumar D. (2023). A novel paper based loop mediated isothermal amplification and lateral flow assay (LAMP-LFA) for point-of-care detection of buffalo tissue origin in diverse foods. J. Food Saf..

[176] Jawla J., Kumar R.R., Mendiratta S.K., Agarwal R.K., Kumari S., Saxena V., Kumar D., Singh P., Boby N., Rana P. (2021). Paper-based loop-mediated isothermal amplification and lateral flow (LAMP-LF) assay for identification of tissues of cattle origin. Anal. Chim. Acta.

[177] Choopara I., Suea-Ngam A., Teethaisong Y., Howes P.D., Schmelcher M., Leelahavanichkul A., Thunyaharn S., Wongsawaeng D., deMello A.J., Dean D. (2021). Fluorometric Paper-Based, Loop-Mediated Isothermal Amplification Devices for Quantitative Point-of-Care Detection of Methicillin-Resistant Staphylococcus aureus (MRSA). ACS Sens..

[178] Xu Z., Yin K., Ding X., Li Z., Sun X., Li B., Lalla R.V., Gross R., Liu C. (2021). An integrated E-Tube cap for sample preparation, isothermal amplification and label-free electrochemical detection of DNA. Biosens. Bioelectron..

[179] Choi J.R., Hu J., Tang R., Gong Y., Feng S., Ren H., Wen T., Li X., Wan Abas W.A.B., Pingguan-Murphy B. (2016). An integrated paper-based sample-to-answer biosensor for nucleic acid testing at the point of care. Lab. A Chip..

[180] Nanayakkara I.A., Cao W., White I.M. (2017). Simplifying Nucleic Acid Amplification from Whole Blood with Direct Polymerase Chain Reaction on Chitosan Microparticles. Anal. Chem..

[181] Emaus M.N., Anderson J.L. (2020). Simultaneous cell lysis and DNA extraction from whole blood using magnetic ionic liquids. Anal. Bioanal. Chem..

[182] Raeber G.P., Lutolf M.P., Hubbell J.A. (2005). Molecularly Engineered PEG Hydrogels: A Novel Model System for Proteolytically Mediated Cell Migration. Biophys. J..

[183] Phelps E.A., Enemchukwu N.O., Fiore V.F., Sy J.C., Murthy N., Sulchek T.A., Barker T.H., García A.J. (2012). Maleimide Cross-Linked Bioactive PEG Hydrogel Exhibits Improved Reaction Kinetics and Cross-Linking for Cell Encapsulation and In Situ Delivery. Adv. Mater..

[184] Xu L., Brito I.L., Alm E.J., Blainey P.C. (2016). Virtual microfluidics for digital quantification and single-cell sequencing. Nat. Methods.

[185] Yi C., Luo Z., Lu Y., Belwal T., Pan X., Lin X. (2021). Nanoporous hydrogel for direct digital nucleic acid amplification in untreated complex matrices for single bacteria counting. Biosens. Bioelectron..

[186] Hügle M., Behrmann O., Raum M., Hufert F.T., Urban G.A., Dame G. (2020). A lab-on-a-chip for free-flow electrophoretic preconcentration of viruses and gel electrophoretic DNA extraction. Analyst.

[187] Persat A., Marshall L.A., Santiago J.G. (2009). Purification of Nucleic Acids from Whole Blood Using Isotachophoresis. Anal. Chem..

[188] Futai N., Fukazawa Y., Kashiwagi T., Tamaki S., Sakai R., Hogan C.A., Murugesan K., Ramachandran A., Banaei N., Santiago J.G. (2022). A modular and reconfigurable open-channel gated device for the electrokinetic extraction of cell-free DNA assays. Anal. Chim. Acta.

[189] Rosenfeld T., Bercovici M. (2018). Amplification-free detection of DNA in a paper-based microfluidic device using electroosmotically balanced isotachophoresis. Lab. A Chip..

[190] Sullivan B.P., Bender A.T., Ngyuen D.N., Zhang J.Y., Posner J.D. (2021). Nucleic acid sample preparation from whole blood in a paper microfluidic device using isotachophoresis. J. Chromatogr. B.

[191] Bender A.T., Borysiak M.D., Levenson A.M., Lillis L., Boyle D.S., Posner J.D. (2018). Semiquantitative Nucleic Acid Test with Simultaneous Isotachophoretic Extraction and Amplification. Anal. Chem..

